# Computational and structural based approach to identify malignant nonsynonymous single nucleotide polymorphisms associated with CDK4 gene

**DOI:** 10.1371/journal.pone.0259691

**Published:** 2021-11-04

**Authors:** Rahatul Islam, Mashiur Rahaman, Hammadul Hoque, Nazmul Hasan, Shamsul H. Prodhan, Asfia Ruhama, Nurnabi Azad Jewel

**Affiliations:** Department of Genetic Engineering and Biotechnology, School of Life Sciences, Shahjalal University of Science and Technology, Sylhet, Bangladesh; King Faisal Specialist Hospital and Research Center, SAUDI ARABIA

## Abstract

Cycline-dependent kinase 4 (CDK4), an enzyme of the cycline dependent or Ser/Thr protein kinase family, plays a role in cell cycle progression (G1 phase) by phosphorylating a tumor suppressor protein called pRB. Alteration of this enzyme due to missense mutation/ nonsynonymous single nucleotide polymorphisms (nsSNPs) are responsible for various types of cancer progression, e.g. melanoma, lung cancer, and breast cancer. Hence, this study is designed to identify the malignant missense mutation of CDK4 from the single nucleotide polymorphism database (dbSNP) by incorporating computational algorithms. Out of 239 nsSNPs; G15S, D140Y and D140H were predicted to be highly malignant variants which may have a devastating impact on protein structure or function. We also found defective binding motif of these three mutants with the CDK4 inhibitor ribociclib and ATP. However, by incorporating molecular dynamic simulation, our study concludes that the superiority of G15S than the other two mutants (D140Y and D140H) in destabilizing proteins nature.

## Introduction

Melanoma, particularly malignant melanoma, is a form of skin cancer caused by pigment-producing cells called melanocytes. Although the etiology of the disease is fully complex including different types of genomic alteration in some genes, e.g. MC1R, CDKN2A, and CDK4, nonfunctional CDK4 is the cardinal hallmark of the disease cutaneous malignant melanoma-3 (CMM3). Furthermore, a vast majority of human tumors are now believed to be occurred due to the deregulation of the CDK4/6–cyclin D–INK4–RB pathway [1–3]. Besides, the interference of the CDK4 gene in tumor progression was disclosed by the observations that repression of CDK4 can contribute to terminal differentiation of erythroleukemia cells, whereas overexpression of CDK4 leads to tumorigenesis of different assortment of cancers inluding glioblastomas and sarcomas, lung cancer, and breast cancers [4–7]. However, germline mutations in the CDK4 are quite rare, having recently been discovered in families of hereditary malignant melanoma. Four such cases are known to date where Arg encoded by the 24 codon is converted to either Cys or His after mutations occur [8–10].

The gene (CDK4) positioned on the long arm of 12(q) chromosome is 5 kb long and has 8 exons, one of which is a non-coding exon [10, 11]. The protein product of this gene is a major component of the protein kinase complex essential for the progression of the G1 phase of cell cycle as it phosphorylates and inhibits pRB protein of retinoblastoma gene [12, 13]. The phosphorylation of pRB enables the transcription factor E2F1 to dissociate from the pRB/E2F1 complexes, and eventually, transcription of E2F1 target genes induces G1 phase progression. [13]. However, CDK4 alone cannot phosphorylate pRB protein and enzyme activity requires both phosphorylation at Thr-172 and binding to a D-type cyclin for activation [14].

The daunting task of cancer studies is to discover the effects of SNPs specifically nonsynonymous single nucleotide polymorphisms (nsSNPs) or missense mutations in the coding sequence of tumor suppressor genes. A single nucleotide polymorphism, or SNP, is known as a single nucleotide variation (e.g., A > T / G / C) at DNA level. SNPs (Single Nucleotide Polymorphism) were also estimated to be responsible for more than 90 per cent of sequence variations in the human genome [15]. However, non-synonymous SNP (nsSNPs) are prioritized mainly because of their involvement in most of the human genetic diseases and their role in disease diagnosis as a biomarker [16]. Furthermore, these nsSNPs may induce amino acid substitutions in the protein sequence which can cause destabilizing conditions of the protein, including loss of stability or interaction between proteins [17, 18].

Current advances in human genome science have yielded a wealth of evidence showing tens of millions of human genetic variations across populations, including SNPs. Study of these variations can provide a framework for analyzing the relevance of these variations in disease susceptibility as well as formulating newer treatments. However, analysis of all these substitutions with the help of laboratory-based techniques is time-consuming, costly, and laborious. An effective alternative to this is the use of methods (*In silico*) based on the biochemical intensity of the amino acid substitution, as well as the protein sequence and/or structural information. Based on this information, various *in silico* approaches have been developed recently to sort out the functional malignant nsSNPs in the candidate protein by utilizing different prediction algorithms. Functional nsSNPs in various genes like PPARG [19], BCL11A [20], CDK7 [21], STN1 [22], BRAF [23] and BRCA1 [24, 25] were thus successfully identified from a broad range of SNP datasets.

In a sense, discovering the effects of nsSNPs in the coding region of the tumor suppressor gene from a large set of data is a challenging task in cancer studies. Thus, this study was conducted with a view to identify the most malignant nsSNPs in the CDK4 genome and further evaluation of the mutants to assess the structural impact of these substitutions on protein structure or interaction. Analysis of this study will aid in shortening of the expensive experimental cost of identifying disease associated SNPs in CDK4 associated diseases for which specialized personalized treatment or therapy can be formulated.

Concisely, in this study we identified the most malignant nsSNPs in the CDK4 genome by approaching various SNP/sequence analysis webservers and computational biology tools, e.g. molecular docking or molecular dynamic simulation. Initially, we sorted out the most malignant nsSNPs from large dataset with some filtration approach by incorporating these webservers. Finally, we analyzed those sorted nsSNPs for structural impact analysis through molecular docking and dynamic simulation. So, finding of this study conclude a detailed computational overview of the disease associated nsSNPs in the CDK4 genome, which may provide a complete blueprint for further wet lab based experiments.

## Methodology

### Collection of SNP’s dataset

The SNP data of CDK4 gene was adopted from the NCBI (National Centre for Biotechnology Information Website) database dbSNP (https:/www.ncbi.nih.gov/snp/) [26]. The sequence of amino acid of CDK4 gene product, a cyclin-dependent kinase protein was obtained from the database of Uniprot (**Uniprot ID: P11802**) [27].

### Assessment of webservers for finding malignant variants in 1^st^ filtration

Distinguishing the malignant and benign forms of SNP from an ample data pool is an intricate process. Nevertheless, a huge number of computational bioinformatics webservers have recently been developed to discern the effect of single amino acid polymorphism or SNPs in a protein. We used those webservers in this analysis to predict the damaging/deleterious/pathogenic SNPs as malignant mutations in the CDK4 gene. Because analyzing the harmful variants by all these different algorithms of webservers added reliability by cross-checking the predicted results; we utilized PROVEAN, SIFT, SNAP2, FATHMM, PON-P2 and Predict SNP as a 1^st^ filtration webservers to predict malignant nsSNPs in the CDK4 protein coding region. SIFT; SNAP-2 and PROVEAN yield results in either tolerated or harmful categories whereas PON-P2 allocates variants into 3 groups: pathogenic, neutral, or unknown classes. SIFT algorithms (https://sift.bii.a-star.edu.sg/), being the first choice in SNP characterization, are often used to predict harmful nsSNPs in almost all computational work [28]. To identify nsSNPs as malignant, SIFT utilizes the homology sequence of proteins with orthologous and paralogous sequences of proteins. A SIFT score of >0.05 is expected to be tolerant while a score of <0.05 indicated harmful effects of nsSNPs on the function or structure of proteins [29]. To predict the malognant variants, the Protein Variation Effect Analyzer (PROVEAN) algorithm (http://provean.jcvi.org/index.php) tool applies delta alignment scores based on the variant version and reference of the protein sequence. In PROVEAN, malignant nsSNPs were characterized by a comparison of the score below the threshold value of −2.5 [30]. Another tool, SNAP2 (https://www.rostlab.org/services/snap/), is focused on advanced machine-learning and neural network based approach to classify the harmful nsSNPs and the functional impacts (effect or neutral) of these variants in protein. To predict the alteration in protein function (gain or loss) it utilizes functional and structural annotations, sequence, and evolutionary qualities of the queried protein. SNAP2 identifies with high accuracy whether the mutation is neutral (ranges from -100 to 0) or effect (ranges from 0 to +100) [31]. PON-P2 (http:/structure.bmc.lu.se / PON-P2/), predicts the mutations of harmful amino acids by means of machine learning algorithms. PON-P2 splits the results into 3 classes: pathogenic, neutral, or unidentified [32]. Another server include FATHMM (http://fathmm.biocompute.org.uk/), which employs Hidden Markov Models (HMM vector) to assess the functional impact of both coding variants, i.e. non-synonymous single nucleotide variants (nsSNVs), and non-coding variants in the human genome [33].

Finally, the Predict SNP server (https://loschmidt.chemi.muni.cz/predictsnp/) can predict neutral or harmful SNPs by integrating these eight predictive tools e.g. MAPP, PANTHER, nsSNP Analyzer, PhD-SNP, PolyPhen-1, PolyPhen-2, SIFT and SNAP. The SNP benchmark dataset in predict SNP is trained on 43,000 unbiased mutations. Thus, by incorporating all the different servers listed above, predict SNP can deliver a significant consensus result with high precision of throughput. The result provided by the predict SNP server falls under two categories, e.g. harmful or neutral, where both categories hold a specific confidence score established on consensus outcome of all the eight different servers mentioned before [34]. However, the accuracy rate extends to a greater extent by incorporating the scores of all the different webservers (PROVEAN, SIFT, SNAP-2, FATHMM, PON-P2 and Predict SNP) and eventually, we sorted out the most malignant mutations associated in CDK4 protein in the 1^st^ filtration.

### Assessment of webservers for finding malignant variants in 2^nd^ filtration

In the **2**^**nd**^ **filtration** process, we utilized 4 different webservers (ModPred, ConSurf, I-Mutant and Mupro) for further sort out the most malignant nsSNPs from CDK4 protein.

ModPred webserver (http://www.modpred.org/) was used to identify the Post translational modification sites (PTM) of each residues in CDK4 protein [35]. PTM sites play a key role in maintaining protein-protein interaction and protein folding after translation. If a residue has a role in Post translational modification sites, then changing of this residue can be malignant for the protein. However, ModPred uses 34 models of logistic regression sets to estimate 23 forms of post-translation modification sites in a protein sequence. The amino acid sequences of native CDK4 protein used as target to identify the post translational modification sites at each residues of the protein.

ConSurf webserver (https://consurf.tau.ac.il/) was employed to predict the conservation of each residue of a protein in the evolutionarily conserved region [36]. Conservation results from consurf webserver is classified into three categories: variable (score: 1–4), intermediate (score: 5–6) and conserved (score: 7–9) [37, 38]. So, alteration of residues in conserved regions can be malignant for protein. Amino acid sequence of CDK4 protein was inserted as a query to find out the predicted conserved patterns based on the conservation score.

We also utilized I-Mutant and Mupro webserver (http://gpcr2.biocomp.unibo.it/cgi/predictors/I-Mutant3.0/I-Mutant3.0.cgi) to predict the protein stability due to the alteration of the non-synonymous residues in the CDK4 protein. If a new residue decreases protein’s stability then this substitution can be clarified as malignant. I-Mutant is a "SVM" approach focused on machine learning for predicting the free energy of a protein due to the specific substitution [39]. The expected free energy change (ΔΔG) is calculated by subtracting the free energy change of the mutant protein (unfolding) from the free energy value of the native protein (unfolding). DDG > 0 indicate that the mutant is stable whereas DDG < 0 clarify the unstable nature of the mutant protein [39]. Finally, we also used Mupro server (https://www.ics.uci.edu/~baldig%20/%20mutation.html) (supporting vector-based machine approach) along with I-Mutant for stability prediction to emphasize the final results [40].

### Modeling and validation of the native and mutant protein structure

Amino acid substitution has a structural impact on the mutant protein and can alter the protein’s stability and functions (Secondary structure, tertiary structure, ligand binding and non-bond interaction). As a result, after identifying the final nsSNPs from 2^nd^ filtration, we developed mutant protein’s 3D model structures along with the native protein to investigate the differences in structure, function and stability. The Robetta server (https://robetta.bakerlab.org/) was used to produce the mutant and native protein’s 3D model structure [41]. Further, ModRefiner (https://zhanglab.ccmb.med.umich.edu/ModRefiner/) was used for energy minimization and refinement of the mutant proteins to increase their structural accuracy [42]. We have also used the structural validation server PROCHECK (https://servicesn.mbi.ucla.edu/PROCHECK/) for validating the designed proteins [43]. In the end, Discovery Studio was used for visualizing the 3D structures of the modeled proteins [44].

### Prediction of structural effect upon mutation

After modeling of the native and mutant protein models, we utilized three different webservers to predict the structural effect of the final nsSNPs in the protein structure. First of all, we utilized the Project Hope server (https://www3.cmbi.umcn.nl/hope/) to characterize the substitution impact of residues in a protein. This server collects structural information including sequence annotations and tertiary protein structure which are readily accessible in the Uniprot database. Project Hope server offers valuable information on the structural changes between the residues of native and mutant proteins by integrating this information [45]. The protein sequence and substitution information (nsSNPs) of CDK4 was used as an input in the Project Hope server for structural effect analysis.

After substitution impact analysis, secondary structure analysis is conducted to predict secondary structure of the mutant and native protein models e.g. alpha helices, beta sheets, and coil regions. We used the STRIDE program (http://webclu.bio.wzw.tum.de/stride/) to evaluate the secondary structural elements of the native and mutant proteins [46]. STRIDE server used the protein’s atomic coordinates and determines the secondary structure by combining hydrogen bond energy with structurally derived information of torsional angle [46]. The STRIDE web interface provides an interactive framework to examine the typical secondary structure across the STRIDE platform. The PDB data were used in the Stride web interface as raw input for secondary structural analysis.

Finally, structural deviations of the mutant models compare to the native was evaluated by calculating the Root Mean Square Deviation (RMSD) score after superimposition. We used the TM-align (https://zhanglab.ccmb.med.umich.edu/TM-align/) (server for protein structural alignment) for superimposition of the mutant proteins with native. TM-align combine the TM-score rotation matrix and Dynamic Programming (DP) to predict the best structural alignment between protein pairs. The server also measures the total RMSD score of the superimposed mutant and native protein models [47].

### Protein-ligand docking and non-bond interaction analysis

Molecular docking is an *in silico* approach that can evaluate the binding correlation between a ligand and a receptor molecule. Protein-ligand docking analysis was conducted between the mutant and native models of the CDK4 protein with ribociclib (A FDA approved inhibitor of CDKs) and ATP. Ribociclib can compete with ATP for binding in the ATP binding cleft of the proteins (CDK4/CDK6) and thus inhibit its activity [48, 49].

We used the PatchDock server (https://bioinfo3d.cs.tau.ac.il/PatchDock/) as molecular docking tool for docking analysis [50]. At first, the receptor (native and mutant proteins) and ligand molecules (ribociclib and ATP) were energy minimized and then docked by maintaining the proper parameters (Clustering RMSD 4 Å, Complex type as protein and small molecule) in PatchDock server. However, The 3D structure of the drug and ATP were retrieved from the PubChem database (PubChem CID: 44631912 for ribociclib and 5957 for ATP) and energy minimized by using MM2 force field method in Avogadro program [51].

After docking, the Patchdock server resolved top 20 best complex solutions of ligand and receptor with highest score, where the best solution was selected having the highest geometric score which was then aligned and visualized in Discovery Studio [44]. In addition, the non-bond interactions between the points of mutational residue with surrounding residues were assessed using the Discovery Studio Visualizer [44].

### Molecular dynamics simulation

MD simulations were executed to unravel the atomic-level changes in the WT-CDK4 protein and its other variants with respect to time. The simulations of the protein models were executed with simulation package GROMACS [52] (version 2020.04) with CHARMM36 force field [53] on a system running the UBUNTU 20.04 OS.

The protein atoms were restrained in a cubical box (dimension: 10Å) solvated withSPC/E (Simple Point Charge) water molecules and periodic boundary conditions wereoptimized to carry out the simulations. Two sodium ions were added to the system to neutralize it. To achieve a consistent conformation, the energy minimization was carried out using 5000 iterations of steepest descent algorithm. Constant number of particles, temperature, and pressure (NPT) ensembles through Parrinello–Rahman biostat and constant number of particles, volume, and temperature (NVT) ensembles through Berendsen thermostat with no pressure coupling were used to equilibrate the energy minimized systems for 100 ps at a constant temperature of 300 K and a constant pressure of 1 atm.

Finally, the systems were subjected to a MD simulation of 100 ns. From the simulation trajectories, RMSD (Root-mean-square deviation), RMSF (Root Mean Square Fluctuation), SASA (Solvent-Accessible Surface Area), Rg (Radius of gyration), and number of Hydrogen bond analysis were carried out. The resulting plots for these analyses were created using the XMGRACE [54] program.

## Results

**Fig 1:**
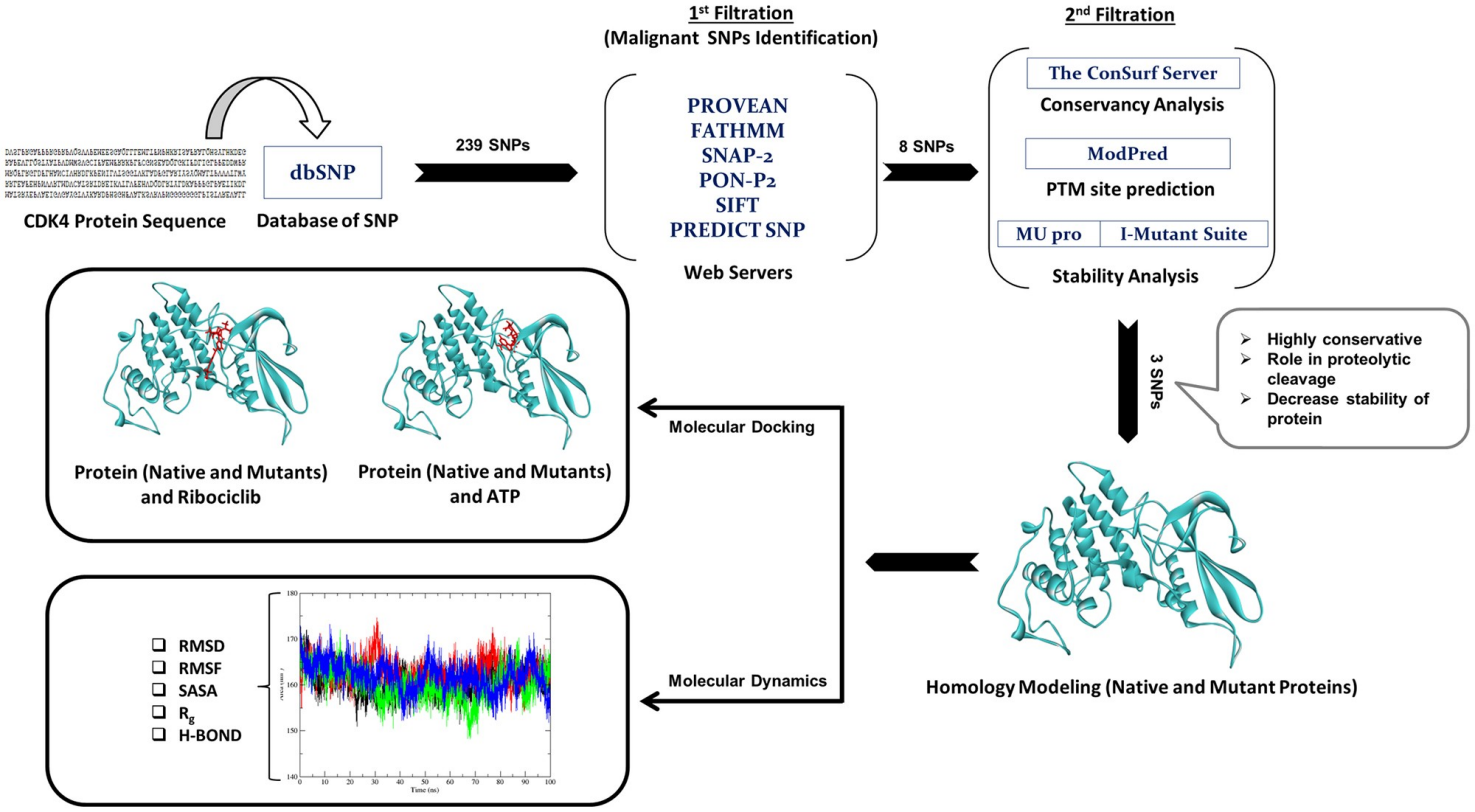
Flowchart for methodology.

The comprehensive workflow and webservers used in human CDK4 to identify the malignant SNPs are summarized in (Fig 1).

### Retrieval of nsSNPs substitutions

DbSNP database holds all the necessary information about CDK4 gene polymorphism. The dbSNP database with CDK4 reported total 2118 SNPs where 852 were found in the intronic region, 239 are nsSNPs (missense), 152 are synonymous, and the rest belongs to other categories. In our study, only the nsSNPs were exposed to further investigation since their interruption may encode altered structure or function of the protein.

### Prediction of malignant mutants in 1^st^ filtration

To sort out the Malignant nsSNPs (Deleterious/Pathogenic/Damaging) from 239 nsSNPs, first of all we utilized six different webservers (PROVEAN, SIFT, SNAP-2, FATHMM, PON-P2 and Predict SNP) as a **1**^**st**^ **filtration process** which is given below.

Initially PROVEAN server was used to evaluate the 239 nsSNPs of CDK4 protein. A total of 112 nsSNPs from this analysis were found as malignant. After we selected those 112 nsSNPs for screening via other databases (S1 Table). According to SIFT algorithm, of the 112 nsSNPs, 89 nsSNPs were identified as damaging and rest of 22 were tolerant. SNAP-2 another webserver also identified 77 nsSNPs having impact on the function of the protein. Even then, to verify the whole result we also analyzed these 112 nsSNPs through multiple sets of in-silico prediction pipelines e.g.: FATHMM, PON-P2 and Predict SNP server. In PON-P2, we found 29 nsSNPs as pathological; only 9 to be damaging in FATHMM; and 66 nsSNPs were deleterious from Predict SNP webserver.

After identifying the malignant nsSNPs from six different webservers, we intersect the prediction of all webservers based on their results and scores. Thus, by combining the algorithm of all six different webservers, finally, eight nsSNPs were identified as highly malignant based on their predictive consensus scores (S2 Table) and selected for further **2**^**nd**^ **filtration process.**

### Prediction of malignant mutants in 2^nd^ filtration

In the **2**^**nd**^ **filtration** process, first of all we utilized the ModPred webserver to predict the Post translational modifications (PTM) sites of all residues of CDK4 protein. ModPred webserver results in only four proteolytic cleavage sites (G15S, D140Y, H132L, and D140H) among the eight highly malignant nsSNPs. However, rests of the four residues were found to have no function in Post translational modification of the protein (S3 Table).

In the consecutive **2**^**nd**^ **filtration** process, we used the ConSurf webserver to assess the conservation of the malignant nsSNPs found from preceding analysis (1^st^ filtration). Evolutionary conserved protein residue is usually considered as malignant when compared with residues in non-conserved position. Analysis of ConSurf webserver indicated highly conserved nature of the eight nsSNPs with highest conservancy score e.g. 9 (S1 Fig).

In the final **2**^**nd**^ **filtration** process, impact of nsSNPs in the stability of CDK4 protein was predicted by I-Mutant and MUpro webserver. Output of these webservers resolved, of the eight highly malignant nsSNPs, five (G15S, D140Y, D140H, G13R, G201D and H132L) were likely to decrease protein stability. Rest of the three nsSNPs (G13V, H132L and P183L) can increase stability of the protein predicted by these webservers (S4 Table).

By combining the prediction of all webservers used in the **2**^**nd**^ **filtration** process, finally we sorted out the final three malignant nsSNPs (G15S, D140Y, D140H) which scored highest in all webservers. So, we selected these three nsSNPs for further structural based analysis (Table 1).

**Table 1:** Selection of final nsSNPs for structural analysis based on combination of effect on PTM sites, conservancy and stability analysis in 2^nd^ filtration.

Variant	Effect on PTM sites	Conservancy	Effect on Stability	
			I-Mutant	Mu-PRO
**G15S**	*✓*	Highly Conserved	Large Decrease	Decrease
**D140Y**	*✓*	Highly Conserved	Large Decrease	Decrease
**G13R**	*✘*	Highly Conserved	Decrease	Decrease
**G13V**	*✘*	Highly Conserved	Increase	Decrease
**H132L**	*✓*	Highly Conserved	Increase	Increase
**P183L**	*✘*	Highly Conserved	Increase	Increase
**G201D**	*✘*	Highly Conserved	Decrease	Decrease
**D140H**	*✓*	Highly Conserved	Large Decrease	Decrease

### Comparison analysis of predicted mutants with somatic mutants

From **2**^**nd**^ **filtration** process, finally we sorted out the three malignant nsSNPs (G15S, D140Y, D140H) which scored highest amongst all nsSNPs retrieved from dbSNP database. However, for the same codon positions, somatic mutations are not found so far in D140. But, For G15, two somatic mutations (G15R and G15C) have been documented in COSMIC (Catalogue of Somatic Mutations in Cancer) database. COSMIC represents the world’s largest and most comprehensive resource for exploring the impact of somatic mutations in human cancer [55]. In COSMIC, the two mutations e.g. G15R and G15C showed significant somatic mutations whereas the primary targeted tissue of G15R is soft tissue (causes sarcoma) and the primary targeted tissue of G15C is skin (causes malignant melanoma). However, as a new malignant mutation was found in the same codon position e.g. G15S in our study, we therefore, conducted a cross analysis to identify whether these somatic mutations e.g. G15R and G15C are pathogenic with all the bioinformatics parameters employed in the **1**^**st**^ and **2**^**nd**^ **filtration** of this study. Interestingly, after analysis, our mutation G15S showed almost similar prediction with G15R and G15C in all the webservers (S5 Table). So, it can assume that this prediction could be a validation of the methods employed in this study.

### Modeling and validation of 3D structure of native and mutant proteins

Robetta webserver was used for generating the 3D models of the native and mutant proteins by comparative modeling technique against PDB ID: 3G33 as a template. The kinetic protein CDK4 alone cannot phosphorylate retinoblastoma gene, pRb and cyclin D complex were required for its activation. The PDB ID: 3G33 is the best known crystal structure of the CDK4/Cyclin D complex and Robetta server used this template for designing structures of the mutant and native models. In addition ModRefiner server was utilized to refine the modeled tertiary structures. Then we checked the model proteins utility by Ramachandran plot analysis through PROCHECK server. PROCHECK analysis revealed 92.1% residues in most favored regions; 7.9% residues in additional allowed regions; where no residues in disallowed regions for G15S mutant. Again Ramachandran analysis revealed 90.5% residues in most favored regions; 9.1% residues in additional allowed regions and 0.0% residues in disallowed regions for D140Y mutant whereas 92.1% residues in most favored regions and additional 7.9% in the allowed with no residue in disallowed regions for D140H mutant shown in S2 Fig.

### Analysis of structural effects of mutations

After modeling of the native and mutant proteins, we utilized three webservers (Project Hope, STRIDE and TM align) to identify the structural impact of the three nsSNPs (G15S, D140Y, D140H) in CDK4 protein.

**Fig 2:**
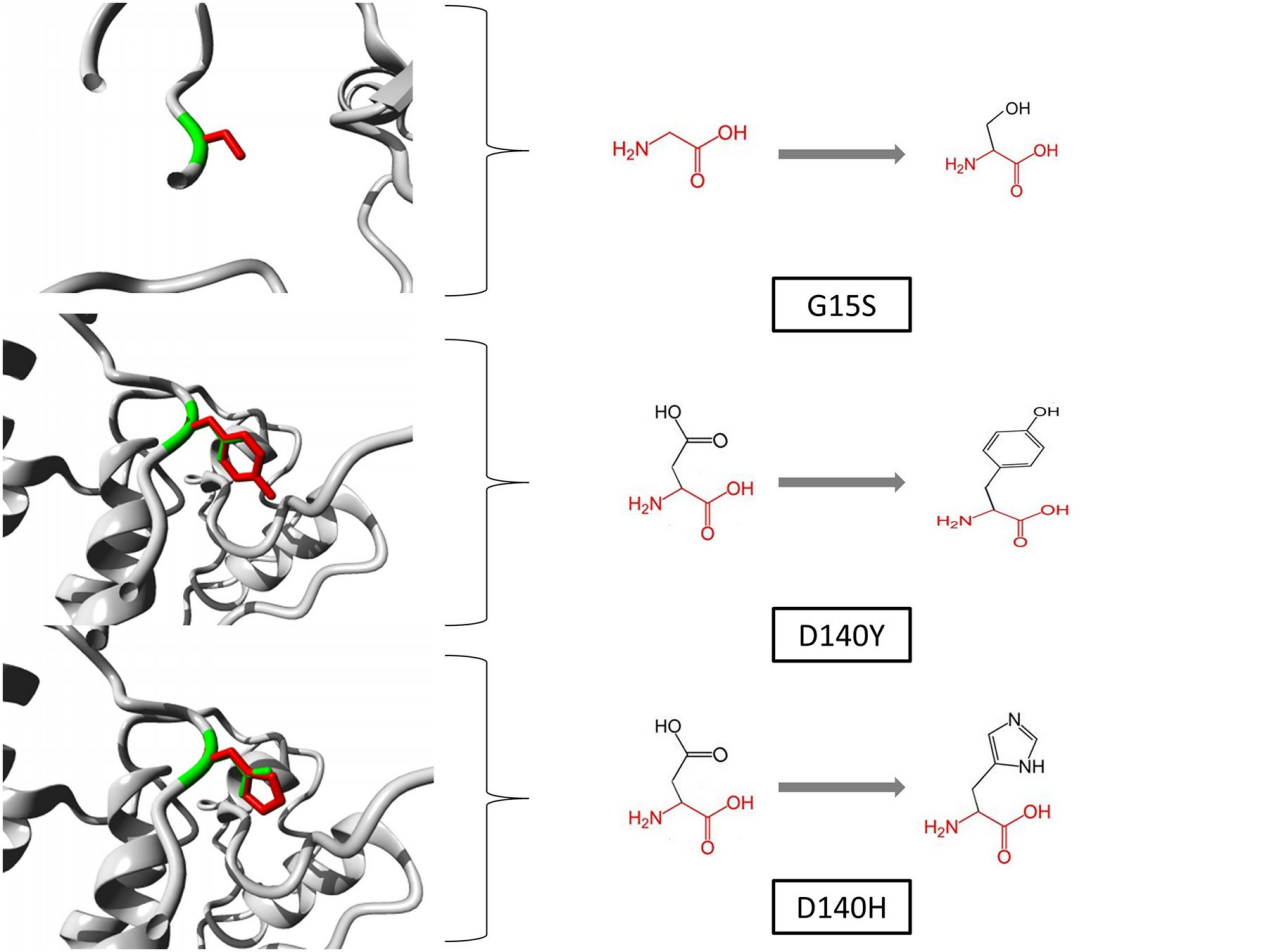
Project Hope server analysis of CDK4 native (green color) and mutant (red color) models to visualize the stereo chemical conformation of wild type and mutant residues at 15th and 140th positions.

Project Hope server’s structural effect analysis showed the replacement impact of serine residue substituted by glycine in the catalytic protein kinase domain of mutant G15S protein. This domain is essential for binding with ATP and other protein-protein interactions (RB1, CDKN2A, CDK6 etc.). However substitution of the buried glycine residue with serine at the 15^th^ position may disturb this domain and can abolish its function. Furthermore, Project Hope server reaffirmed that the native residue is located on the surface of the protein; mutation of this residue can disturb interactions with other molecules or other parts of the protein. Glycine is also extremely flexible and this flexibility may be essential for the proper functioning of the protein. Further in case of both D140Y and D140H mutants; by replacing tyrosine and histidine residue in place of aspartic acid at the 140^th^ position in the protein kinase domain of CDK4 protein can trigger similar unstable conditions predicted by Project Hope server. Moreover, the wild type residue is more hydrophobic than the mutated residues in both cases and the wild-type residue forms a salt bridge with several surrounding residues including R101, K142 and R142. But after mutation the charge differences in mutants may disturb the original residue’s ionic interaction and may cause loss of the salt bridge bonding with other surrounding residues. The stereo chemical conformation of wild type and mutant residues predicted by Project HOPE server are shown in Fig 2.

**Fig 3:**
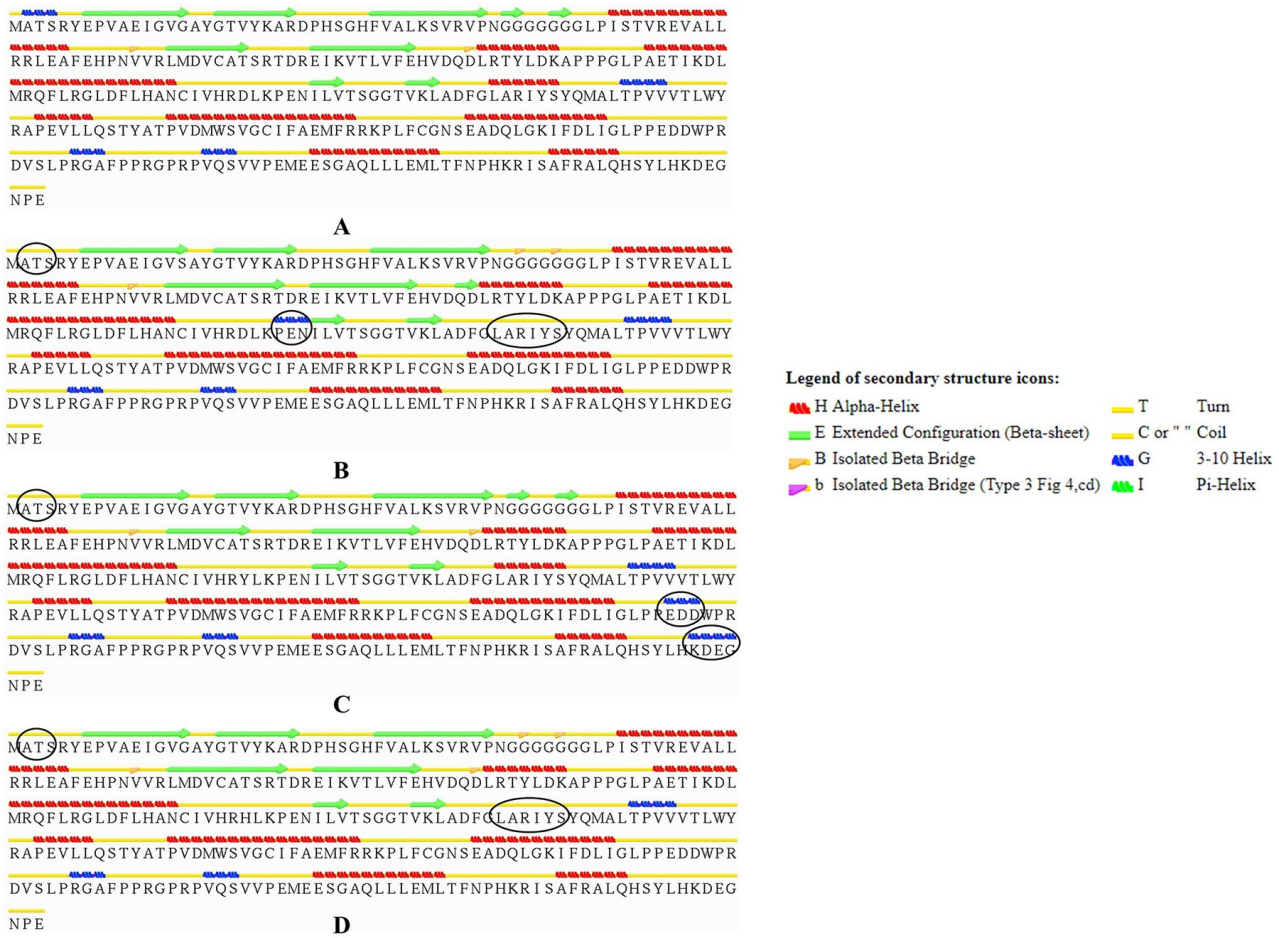
Secondary structure analysis of native and mutants by STRIDE program. Secondary structures elements of (A) native CDK4 (B) Mutant G15S (C) Mutant D140Y and (D) Mutant D140H protein. Changes in Structural elements are shown in circle.

Further, Secondary structure analysis of native and mutant proteins through STRIDE program showed significant alteration in the average secondary structure of the mutant proteins compared to the native (Fig 3). The major alteration was observed in the 310-helix regions (A2-S3) of the native protein where this region is converted to coil (less stable than 310-helix) in all the mutants. Helix and strands improves the stability of the protein. Alteration in the helix region thus can be destructive for both protein structure and function. Furthermore, another prominent helix region (H6) (L161-S166) in native CDK4 was also converted to coil region in both of the mutants G15S and D140H. Contrastingly, in case of D140Y mutant; two coil regions (E235-D237 and K297-G100) were converted to 310-helixes which are a signs of structure improvement. By analyzing secondary structure analysis, G15S and D140H were identified as less stable mutants and can cause structural alteration in the overall protein structure.

**Fig 4:**
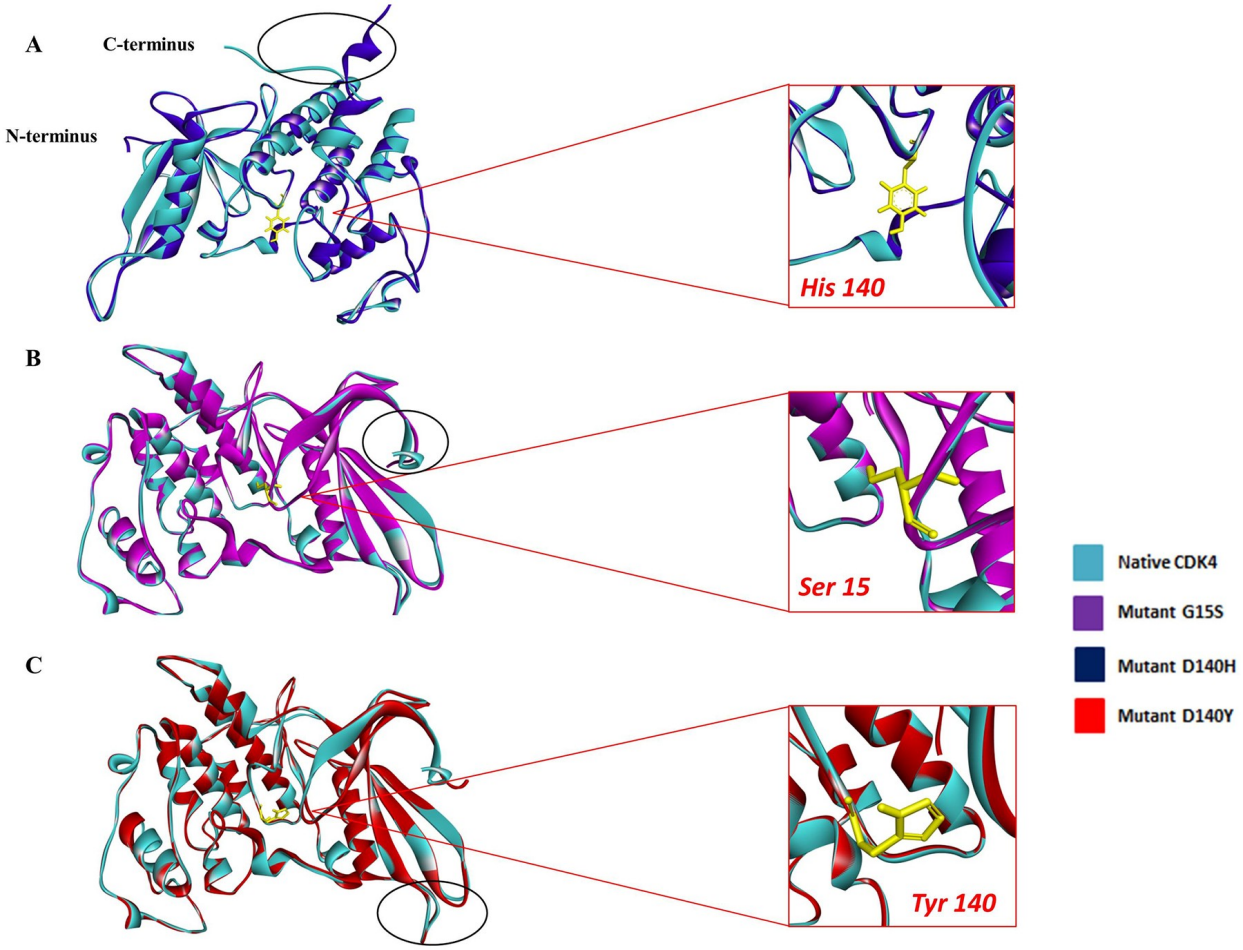
Superimposed structure of native and mutant proteins. (A) Native CDK4 superimposed with D140H mutant (B) Native CDK4 superimposed with G15S mutant and (C) Native CDK4 superimposed with D140Y mutant. Deviation of structural changes is shown in circle. Total RMSD was found 1.32 Å for mutant G15S; 0.94 for mutant D140Y and 1.04 Å for mutant D140H.

Finally, we superimposed the 3D structure of native and mutant proteins to obtain more clear vision of the structural deviation caused by these nsSNPs. The superimposed structure of the native with mutant proteins demonstrated a significant deviation in the mutant structures compared to the native protein CDK4 (Fig 4). Total Root mean square deviation (RMSD) value of the superimposed mutant with native protein was found 1.32 Å for G15S; 0.94 Å for mutant D140Y and 1.04 for mutant D140H by TM align server. Thus, G15S mutant disclosed higher structural deviation compared to the rest two mutants.

### Protein-ligand docking analysis

**Fig 5:**
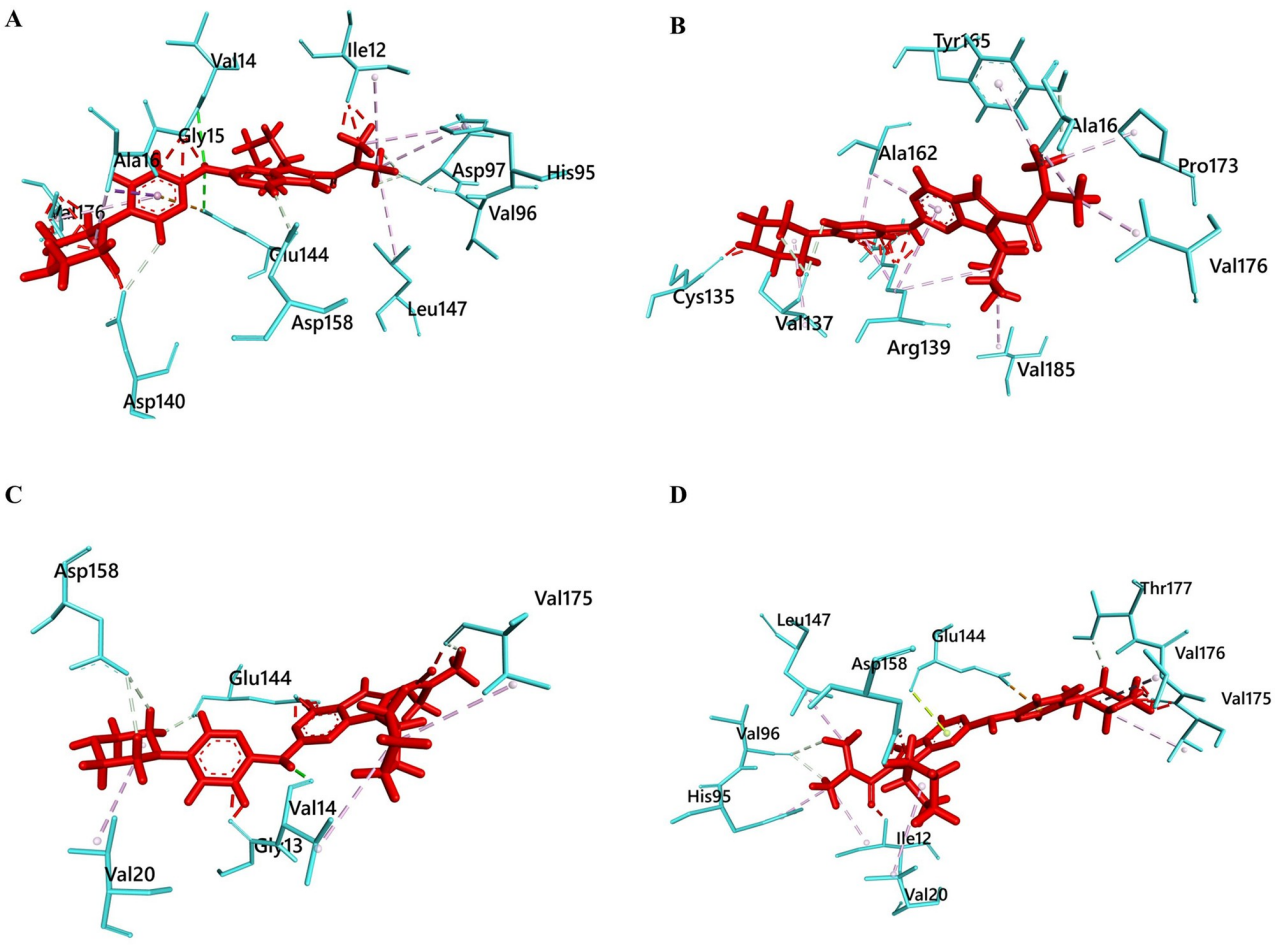
Protein-ligand residual interaction analysis of (A) Native CDK4 (Aqua) with ribociclib (red) (B) G15S mutant (aqua) with ribociclib (red) (C) D140Y mutant (aqua) with ribociclib (red) and (D) D140H mutant (aqua) with ribociclib (red). The interacting bonds are displayed as: Conventional hydrogen bonds/ Carbon hydrogen bonds as green line, pi-pi stack/pi-alkyl as pink lines, Salt bridge/ attractive charge/ electrostatic as brown lines.

Protein-ligand docking analysis was carried out with ribociclib and the tertiary structure of the modeled proteins (Native and mutant both) of CDK4. Alteration in mutated protein structure may affect the binding interaction of ribociclib with CDK4 protein. Studies on CDK4 also revealed some specific residue to be more frequent for binding in the ATP cleft naturally e.g. Ile12, Val20, Ala33, Val77, Phe93, Glu94, His95, Val96, Gln98, Asp99, Thr102, Glu144, Leu147, Ala157, Asp158 whereas Glu144 is the key binding residues which contacts with the terminal phosphates of ATP [56]. Ribociclib competes for these residues by acting as an antagonist of ATP and thus effectively inhibits the kinetic activity of the protein. However our results indicated altered interaction of the ribociclib with mutated models e.g. loss of binding interaction of the ligand with this residues. Patchdock analysis finds out the binding efficiency of the ligand in the form of PatchDock score and atomic contact energy (ACE) values. High patchdock scores and low ACE is expected to be good docked complex and in this analysis we found that native CDK4 protein obtained high PatchDock score and ACE as 5582 and −210.58, respectively. But, all the mutant structures (G15S, D140Y, and D140H) obtained low PatchDock scores and high ACE (D140Y: 4890, -180.76; D140H: 4987, -196.88) except for G15S mutant which showed high Patchdock score of 6582 and ACE as -233.18. However, we studied the contacting residue and bond interaction of the native and mutant proteins with ribociclib and found something more compelling (Fig 5). Results showed new binding cleft of G15S mutant for ribociclib binding whereas the natural ATP binding residues were absent. Thus G15S mutant results in new binding pocket for ATP binding site where the other mutants showed interaction in the same ATP binding pocket with decreased residual contact. These reductions in the number of residue contacts obviously influence the complementarities between mutant protein and ribociclib. Furthermore, the binding interaction of ribociclib with the native protein was formed by total 7 polar (H-bond) and 7 non-polar (Hydrophobic) bonds which were decreased to total nine in both of the mutants (D140Y and D140H). However, these polar bonds and H bonds are crucial in protein structure since they help to maintain the protein ’s stability [57]. Contrastingly in case of G15S mutant the binding interaction of ribociclib was formed by total 3 polar and 12 non-polar bonds in the new binding site of the G15S mutant. Thus the three mutants showed fluctuated binding interaction of the ribociclib with CDK4 which can reduce the inhibitory mechanism of the ribociclib during abnormal cell proliferation. The overall non-bond interactions (H-bond, Hydrophobic, Electrostatic and others) of native and mutant protein complex with ribociclib are shown in Table 2.

**Table 2:** Protein-ligand interaction analysis of native and mutant proteins with ribociclib (H = Hydrogen bond; CH = Carbon Hydrogen bond).

Interacting Residue	Distance (Å)	Bond Types	Interacting Residue	Distance (Å)	Bond Types
**Native CDK4**			**Mutant G15S**		
VAL14	3.0	H	VAL137	2.8	CH
GLU144	2.9	H	TYR165	3.0	CH
ASP158	2.3	CH	VAL137	2.7	CH
VAL96	2.3	CH	ALA16	3.2	Alkyl
ASP97	2.9	CH	ALA16	3.5	Alkyl
ASP97	3.0	CH	ARG139	5.2	Alkyl
ASP140	2.7	CH	VAL185	5.3	Alkyl
GLU144	4.3	Pi-Anion	VAL137	4.4	Alkyl
ALA16	3.3	Pi-Sigma	VAL176	2.7	Alkyl
ALA16	3.1	Alkyl	PRO173	4.5	Alkyl
ILE12	5.0	Alkyl	TYR165	4.1	Pi-Alkyl
LEU147	5.0	Alkyl	ARG139	4.6	Pi-Alkyl
HIS95	5.0	Pi-Alkyl	ALA162	4.5	Pi-Alkyl
HIS95	5.2	Pi-Alkyl	ARG139	4.0	Pi-Alkyl
VAL176	5.0	Pi-Alkyl	ALA162	3.1	Pi-Alkyl
**Mutant D140Y**			**Mutant D140H**		
VAL14	1.3	H	THR177	2.1	CH
ASP158	2.4	CH	VAL96	2.4	CH
GLU144	2.2	CH	VAL96	3.0	CH
ASP158	2.9	CH	GLU144	2.6	Pi-Anion
VAL175	2.6	CH	GLU144	3.0	Pi-Lone
GLU144	2.6	CH	VAL20	4.4	Alkyl
VAL14	5.5	Alkyl	VAL175	5.5	Alkyl
VAL175	4.9	Alkyl	VAL176	5.1	Alkyl
VAL20	5.4	Alkyl	ILE12	3.7	Alkyl
			LEU147	3.5	Alkyl
			HIS95	3.4	Pi-Alkyl

### Protein-ATP docking analysis

**Fig 6:**
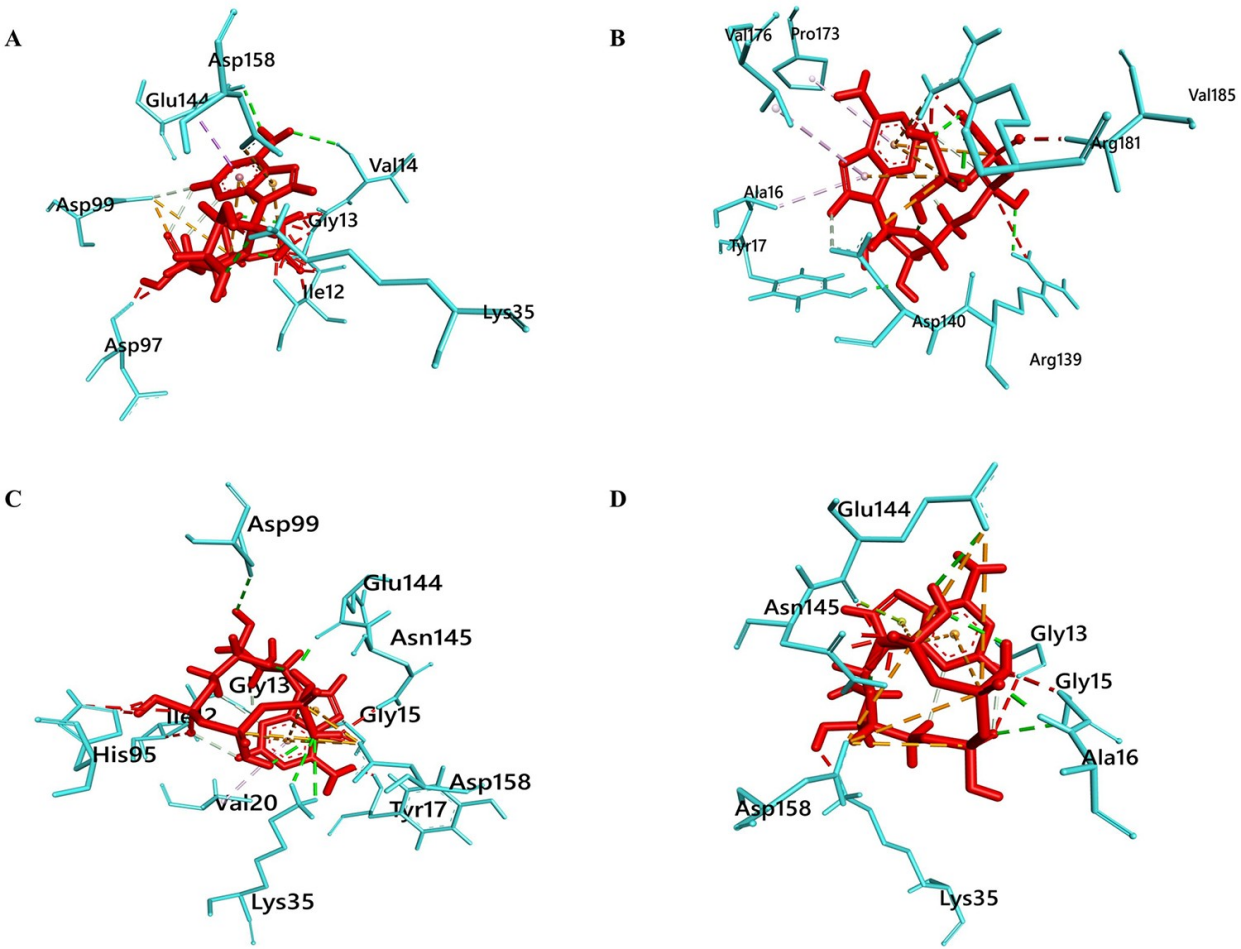
Protein-ligand residual interaction analysis of (A) Native CDK4 (Aqua) with ATP (red) (B) G15S mutant (aqua) with ATP (red) (C) D140Y mutant (aqua) with ATP (red) and (D) D140H mutant (aqua) with ATP (red). The interacting bonds are displayed as: Conventional hydrogen bonds/ Carbon hydrogen bonds as green line, pi-pi stack/pi-alkyl as pink lines, Salt bridge/ attractive charge/ electrostatic as brown lines.

To further evaluate the ATP binding affinity of the mutant models we docked ATP with native and mutant proteins which resolved significant differences in binding affinity. Docking analysis of ATP with Native protein by Patchdock server revealed lowest Patchdock score of 4382 where the highest score was observed in mutant G15S e.g. 5822. Even if the other two mutants also showed larger patchdock score than native which were 4836 for D140Y and 5114 for D140H. This difference indicated the significant binding improvement of the ATP with the mutant protein models. However, residual bond information analysis of the ATP-protein complex further resolved same compelling result as ribociclib where, ATP binds at the same binding pocket of native CDK4, mutant D140Y and D140H models (Fig 6). However, same as before G15S-ATP complex showed binding of the ATP in the new binding cleft with altered bond interaction e.g. no interaction with the natural ATP contacting residues of the CDK4 protein. In addition, ATP interaction analysis with native and mutant protein showed an increasing number of Polar, Non polar and Electrostatic charge interactions (non-covalent interactions) of ATP with residues in mutant proteins compared to the native. This metadata concludes prominent perturbation of the ATP binding site in the G15S mutant while the other two mutants showed significant improvement which may affect the protein by increasing hyper activity against the ATP during overexpression. Thus this deviation observed in the bound ATP molecule can lead to an altered catalytic efficiency of CDK4 by significant loss of ATP binding site in G15S mutant whereas the former two mutants can cause overexpression of the protein. Overall non-bonded interactions of native and mutant protein complex with ATP are shown in Table 3.

**Table 3:** Protein-ligand interaction analysis of native and mutant proteins with ATP ((AC = attractive electrostatic charge interaction; H = Hydrogen bond; CH = Carbon Hydrogen bond).

Interacting Residue	Distance (Å)	Bond Types	Interacting Residue	Distance (Å)	Bond Types
**Native CDK4**			**Mutant G15S**		
ASP99	3.9	AC	ASP140	4.0	AC
ASP99	4.9	AC	TYR17	1.7	H
LYS35	2.6	H	ARG139	2.4	H
VAL14	2.8	H	ARG181	2.0	H
GLU144	2.4	H	ASP140	2.8	CH
ASP99	2.6	CH	ARG181	4.7	Pi-Cation
ASP158	4.9	Pi-Anion	ALA16	4.2	Pi-Alkyl
GLU144	3.9	Pi-Sigma	VAL176	5.0	Pi-Alkyl
			PRO173	5.2	Pi-Alkyl
**Mutant D140Y**			**Mutant D140H**		
ASP158	4.9	AC	ASP158	4.4	AC
ASP158	3.0	AC	GLU144	5.6	AC
LYS35	2.6	H	ASP158	4.8	AC
LYS35	3.0	H	GLU144	4.5	AC
ASP99	2.8	H	ASP158	4.3	AC
GLU144	2.0	H	GLY15	2.8	H
GLY15	3.2	CH	ALA16	1.8	H
GLY13	1.9	CH	GLU144	2.8	H
ASP158	4.5	Pi-Anion	ASP158	2.1	CH
ASP158	4.5	Pi-Anion	GLU144	2.8	Pi-Lone
VAL20	4.2	Pi-Alkyl			

### Non-bonding interaction analysis

**Fig 7:**
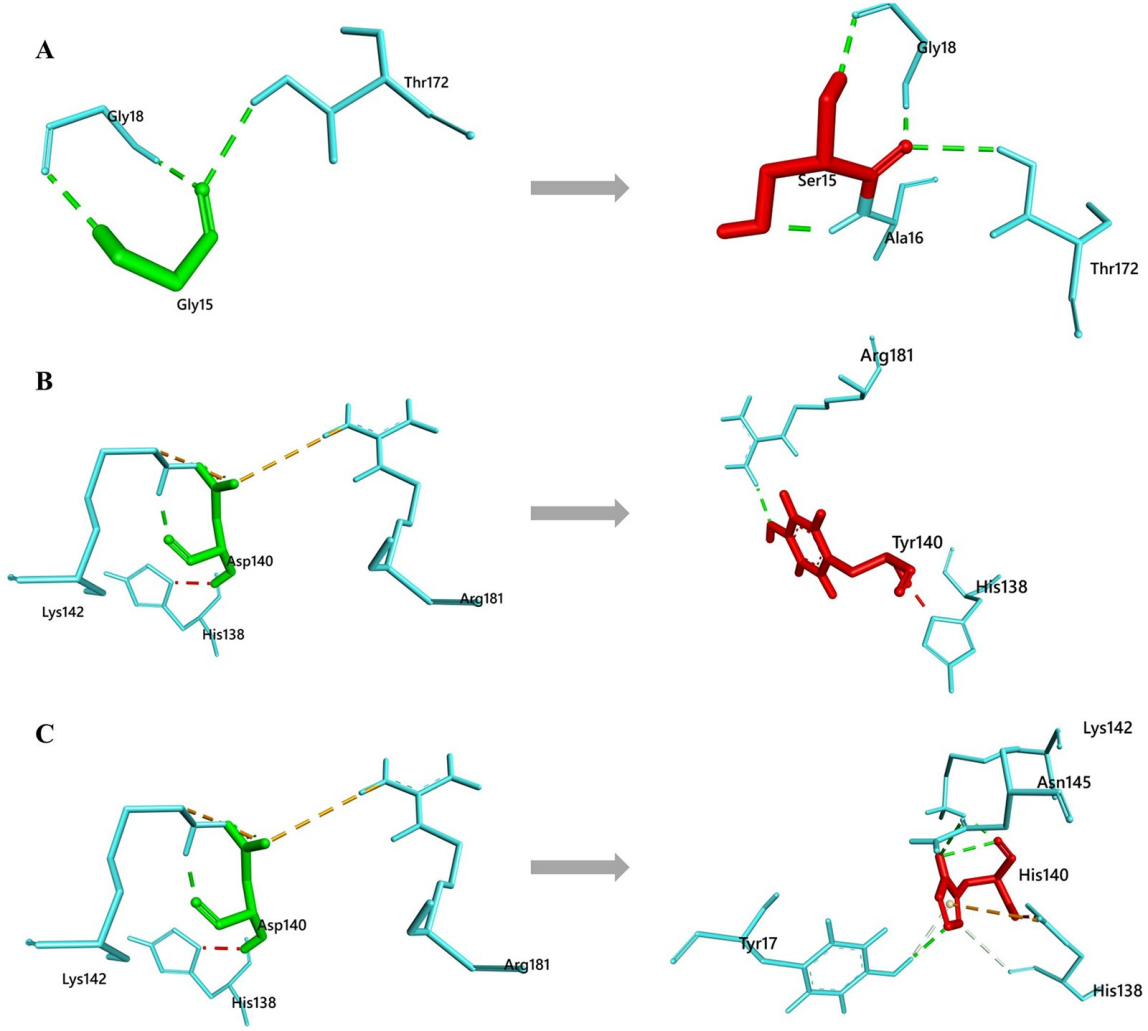
Non-bonding residual interaction analysis of Native and Mutant CDK4 proteins (A) Native Gly15 and mutated Ser15 (B) Native Asp140 and mutated Tyr140 and (C) Native Asp140 and mutated His140. The interacting bonds are displayed as: Conventional hydrogen bonds/ Carbon hydrogen bonds as green line, pi-pi stack/pi-alkyl as pink lines, Salt bridge/ attractive charge/ electrostatic as brown lines.

To determine the interaction of native and mutated residues with surrounding residues non-bonding interaction were further analyzed. Analysis resolved consequent reduced interactions with the surrounding residues only in the Tyrosine residue at 140th position (Fig 7). This reduction is mainly caused due to the inability of the mutant residue (Tyrosine) to form the 3 H-bond interaction with the Lys142 found in the native protein (Table 4). However the non-bonding interaction of the G15S and D140H mutant showed increased binding contacts with surrounding residues (Fig 7). Native G15 showed 3 H-bond with surrounding residues (Gly18, Gly18 and Thr172) whereas mutant 15S residue reported four H-bond with surrounding residues (Gly18, Ala16, Gly18 and Thr172). Also in case of D140 native residue, number of interaction with surrounding residues were four (Lys142, Lys142, Arg181 and Lys142). But the mutant 140H residue showed increased interaction with surrounding residues by forming five H-bond (Tyr17, Asn145, Lys142, His138 and Tyr17) and one electrostatic bond (His138) (Table 4).

**Table 4:** Non bond interaction analysis of native and mutated residues with surrounding residues (UDD = Unfavorable Donor-Donor clash).

Interacting Residue	Distance (Å)	Bond Types	Interacting Residue	Distance (Å)	Bond Types
**Native Gly15**			**Mutant Ser15**		
GLY18	2.3	H	GLY18	2.3	H
GLY18	2.0	H	ALA16	1.9	H
THR172	2.6	H	GLY18	1.9	H
			THR 172	2.6	H
**Native Asp140**			**Mutant Tyr140**		
LYS142	1.9	H	ARG181	2.0	H
LYS142	2.7	H	HIS138	1.9	UDD
ARG181	5.2	Electrostatic			
LYS142	2.0	H			
			**Mutant His140**		
			TYR17	2.2	H
			ASN145	3.0	H
			LYS142	1.9	H
			HIS138	3.5	H
			HIS138	4.3	Electrostatic
			TYR17	2.9	H

### Molecular dynamics analysis

The effect of nsSNPs on the protein structure of CDK4 was analyzed by molecular dynamics simulation. After the dynamics simulations of the wild-type and other variants of the protein, analyses of the simulations were done for RMSD, RMSF, Rg, SASA and Hydrogen bond numbers.

**Fig 8:**
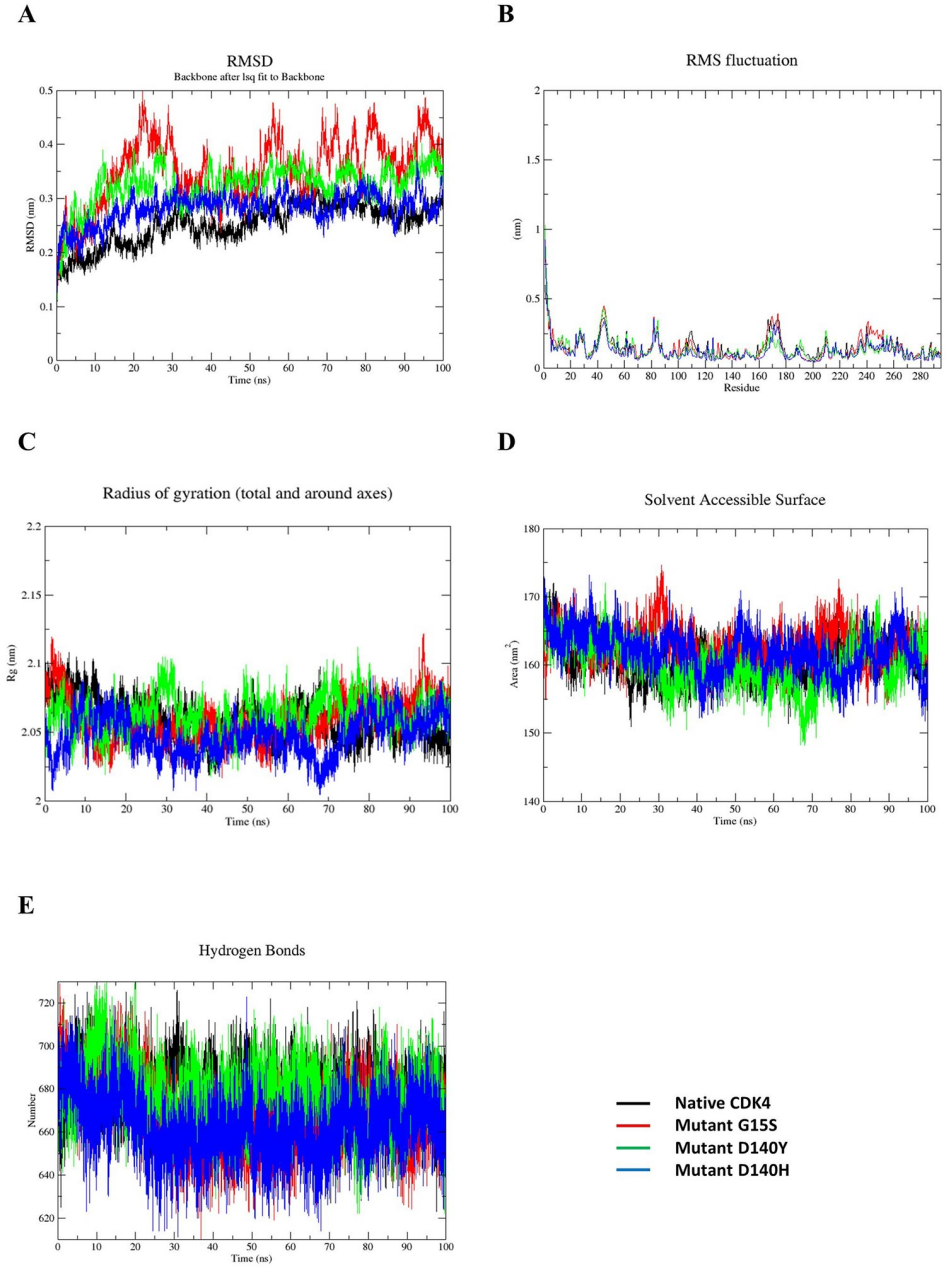
Molecular dynamics analysis (RMSD, RMSF, Rg, SASA and H-bond) of native and mutant CDK4 protein at 100 ns. (A) RMSD values of Cα atoms of native and mutant structures. X-axis is RMSD (nm), and the y-axis is time (ns) (B) RMSF values of the carbon alpha over the entire simulation. X-axis is RMSF (nm), and the y-axis is residue. (C) Rg of the protein backbone over the entire simulation. X-axis is Rg (nm), and the y-axis is time (ps). (D) Solvent accessibility of native and mutant proteins. X-axis is SASA (nm^2^), and the y-axis is time (ns). (E) Total number of H-bond count throughout the simulation of native and mutant structures. The symbol coding scheme is as follows: native (black colour), mutant G15S (red colour), mutant D140Y (blue) and D140H (green colour).

From the overlapping graph (Fig 8A) of the simulations, we can see that there are great deviations (RMSD) in the simulations of the mutant type compared to the wild type. Simulations of all the variants fluctuate greatly from the wild type and the mutant G15S showed the highest deviations of them all. The values of the G15S variant showed higher range compared to all the other variants fluctuating from 0.2nm to 0.5nm whereas the values of the wild type remained in 0.2 to 0.3nm range. This indicates the superiority of G15S variant over all the others in the case of protein instability. Variant D140H showed the least deviations of them all.

The trend was followed in RMSF analysis also. RMSF values of a simulation illustrate the flexibility of a protein structure. G15S variant again showed the greatest deviation particularly in the 240 to 260 residues range. All the other variants also showed higher flexibilities compared to the wild type in the simulation (Fig 8B).

The compactness of the protein is measured by the *R*_*g*_ values of a simulation. In this case, variant D140H showed the biggest variations of them all. It showed reduced number of *R*_*g*_ values throughout the simulation meaning a more compact structure than the others. *R*_*g*_ values of the D140Y remained close to the wild protein during this simulation. Variant G15S showed slightly higher values than the wild protein (Fig 8C).

SASA values of a simulation are associated with the region of a protein that is accessible solvent molecules. SASA values of all the variants were inconsistent with the wild type throughout the simulation. G15S variant showed higher SASA values compared to the wild type whereas the D140Y variants showed lower values than the wild type. These inconsistent SASA values suggest a change in hydrophobic interaction between amino acid residues from the solvent accessible to the buried zone of the variants over the wild type. D140H variant did not deviate too much from the wild type and remained close to the values of the wild type throughout the simulation (Fig 8D).

The hydrogen bond analysis showed substantial changes in the number of intermolecular hydrogen bonds throughout the simulations. For the first 30 ns of the simulation, all the variants did not show significant change in Hydrogen bonding. After that, all three variants showed lesser number of hydrogen bonds forming in the simulation compared to the wild type. This may be attributed to the inclusion of a malignant amino acid. G15S had the least number of Hydrogen bonds forming in the simulation whereas D140Y had the closest number of Hydrogen bonds with the wild type (Fig 8E).

## Discussion

The number of known non-synonymous variants in the human genome databases has over-saturated recently due to the implementation of high-throughput sequencing techniques. Characterization of any of these known natural variants is a time-consuming, labor-intensive, and costly process in terms of their corresponding role to the specific phenotype. In addition, increasing exponential numbers of nsSNPs make it impossible to determine the biological effects of these polymorphisms through laboratory experiments. Conversely, the ability to use computational techniques to differentiate between neutral non-synonymous and pathogenic single nucleotide polymorphisms (nsSNPs) could greatly aid in targeting of disease-associated mutations by filtering out the most likely pathological variants from a large pool of SNP datasets.

Diverse experimental studies have been carried out over the past few years by assessing the relationship between drug response and nsSNPs for cancer treatment. In an apprehending analysis Chambers et al. reported the involvement of nsSNPs in the alteration of protein structure and function [58]. Wang and Moult [59] further delineated the role of nsSNPs in protein-protein interactions, protein expression, stability, folding, alternative splicing, and catalysis or ligand binding that may cause or affect a disease. CDK4 is such a type of gene that almost involves in the development of many types of cancer including melanoma, breast cancer and lung cancer. CDK4 is also associated with a lot of protein including RB1, Smad3, Cdt1, MARCKS, FOXM1, and PRMT5 and several of these proteins have been shown to serve as substrates for CDK4 activity [60]. So, any changes in CDK4 gene cause disruption in many downstream targets of CDK4 protein. However, CDK4 is the member of the broad CDK (Cyclin Dependent Kinase) family or Ser/Thr protein kinase family that can modify the activity of retinoblastoma protein “RB1” during cell cycle progression [60]. It is a catalytic subunit of the protein kinase complex that is required for G1 phase progression in the cell cycle. This kinase’s activity is limited to the G1-S phase, which is regulated by the regulatory components D-type cyclins and the CDK inhibitor p16INK4a [14]. One example is that, Ser/Thr-kinase component of cyclin D-CDK4 (DC) complexes inhibit members of the retinoblastoma (RB) protein family including RB1 by phosphorylation [13]. Phosphorylation of RB1 allows dissociation of the transcription factor E2F from the RB/E2F complexes and the subsequent transcription of E2F target genes which are responsible for the progression through the G1 phase [13]. Thus CDK4 regulates the cell-cycle during G1/S transition.

There are however huge quantities of SNPs that have been detected in human CDK4 genome to date. But all SNPs may have no functional impact on the protein or might have the consequence of low frequency that is not feasible for analysis. The present research, which uses various computational tools, focused on illuminating certain novel CDK4 nsSNPs that may have biological significance for the protein. And yet no single bioinformatics method can be sufficiently accurate to find out the entire blueprint of the functional significance of every gene polymorphism. Therefore, a broad collection of complementary bioinformatics methods have been applied in the current research.

We used six different extensively used computational servers, notably SIFT, PROVEAN, FATHMM, SNAP-2, PON-P2 and Predict SNP in the first line up sorting methods (1^st^ filtration) for identifying nsSNPs in CDK4 protein of the CDK4 gene. The algorithms used in those six servers were theoretically distinct from each other and thus the comparison scores of all those resources would guarantee high prediction reliability. Ultimately eight nsSNPs from 239 nsSNPs of the CDK4 gene were recognized as highly malignant and assigned for further downstream filtration analysis (2^nd^ filtration).

Typically, conserved residues of a protein are more crucial because of the supposition that they are involved in the stability or folding of proteins [61]. We used the Consurf analysis tool in the 2^nd^ filtration to measure the evolutionary conservation profile of all the amino acid positions in CDK4 protein to further identify the most malignant nsSNPs. Consurf’s study confirmed, all of these eight final nsSNPs were strongly conserved and buried, which can be speculated to be located at the protein’s enzymatic sites and indicate important protein-protein interactions. In addition, subsequent study by Modpred server, of these retained residues (eight) for post-translation protein modification sites prediction, participation in proteolytic cleavage was seen only in four nsSNPs. Finally, we used the I-Mutant and MU-Pro as protein’s stability prediction servers in the 2^nd^ filtration process. These side-by-side intersect evaluations in the 2^nd^ filtration process deduced the eight nsSNPs to three (G15S, D140Y and D140H) for further analysis of the structural impact on the CDK4 protein.

After sort listing the nsSNPs from 1^st^ and 2^nd^ filtration, 3D structures of CDK4 proteins (native and three mutants) were designed to obtain the structure level alterations attributable to these adverse polymorphic modulations. To resolve the structure level alteration in protein e.g. structural impact, alteration in secondary structure and structural deviation analysis; again three different web servers were utilized respectably Project HOPE, STRIDE and TM align. The G15S mutant results in substitution from Glycine (a non-polar amino acid) to serine (a polar amino acid) in the protein kinase domain of the protein. Project HOPE server estimated that this larger size mutated residue (serine) is likely to disrupt the interaction with other protein residues in the neighboring region as the native residue (glycine) is located on the surface of the protein. In addition, Project HOPE evaluated the substitution of the buried aspartate residue with tyrosine and histidine at the protein’s 140^th^ position leads to a loss of hydrophobicity in the protein’s core. This loss of hydrophobicity may cause reduction in the ionic interaction with surrounding residues. Thus, these abnormal changes in protein could lead to the loss of thermodynamic stability during aggregation and folding [62].

In addition, secondary structure analysis of the mutant proteins compared to native found major alterations in alpha helix, beta turn, beta sheet and coil regions. For example, in all the mutants; residues of A2-S3 have been completely converted from 310-helix to coil. However, the most prominent modification was found in the mutants G15S and D140H where the secondary structures of residues L161-S166 were fully converted to coil from alpha helix.

Also, the superimposition of mutant model structures with native structure showed more RMSD deviation (1.32 Å) in the G15S mutant than the other two mutants. This metadata concludes that the mutant G15S has more superiority in alteration of the native protein’s 3D structure.

Molecular docking analysis was conducted to further emphasize the supposition as to which mutants have more negative impact on the protein. Molecular docking is a popular *In silico* method which is particularly used in drug designing and vaccine designing approaches to resolve the binding status between a drug/vaccine with its corresponding macromolecules [63, 64]. Ribociclib is such kind of drug (act as CDK4/6 inhibitor) used for treatment of HR+, HER2– advanced or metastatic breast cancer. In our study, we used Ribociclib for docking analysis with CDK4 as it was approved by FDA (U.S. Food and Drug Administration) with a broader indicated patient population in 2017. There are also other drugs are available as CDK4/6 inhibitors e.g. palbociclib and abemaciclib respectively. Palbociclib is the first CDK4/6 inhibitor received FDA approval for HR+, HER2– breast cancer treatment in 2015 whereas abemaciclib is the third approved CDK4/6 inhibitor (approved in 2018) [65]. However, in our study, analysis of the docking showed major binding perturbation of Ribociclib with G15S model’s binding pocket. Whilst less strong binding motif was observed in mutant D140Y and D140H protein compared to the native docked complex, G15S model’s docking analysis found a new binding cleft for Ribociclib binding. That means natural binding pocket for ribociclib with the protein is disrupted in the mutant G15S model. Further, we docked ATP with the native and mutant proteins to resolve the significance of the hypothesis obtained from ribociclib binding. Again ATP binding in the G15S mutant showed new binding cleft interaction whereas the other two mutants (D140H and D140Y) showed interaction in the same binding cleft as native-ATP complex. This further verified that ATP binding affinity can be disrupted in the G15S mutant due to the substitution of single residues in the 15^th^ position of the protein.

We also studied the non-bonding interaction of mutant residues with neighboring residues at the point of mutational position. Results showed reduced interaction only in D140H mutant whereas the other two mutants (G15S and D140Y) showed increased interaction within 4A ° surroundings residues.

Finally, MD simulation analysis was done to assess the behavior of the modeled proteins in a simulated environment based on RMSD, RMSF, Rg, SASA, and Hydrogen bonding. The RMSD analysis revealed the unsteady nature of all the variants compared to wild type. RMSF analysis revealed the increased flexibility of the variants. In both cases, G15S variant showed the greatest deviations. Rg analysis found that the variant D140H had the highest level of compactness throughout the time over all the other variants, whereas G15S showed expansion compared to the wild type. In SASA analysis, all three variants showed great degrees of variability over the wild type and are possibly responsible for changes in protein-protein interactions. Hydrogen bond number analysis did not show a great deviation in the simulations, but variable numbers of Hydrogen bonds were seen to be forming throughout all the time.

Based on simulation studies, it can be hypothesized that the variants G15S, D140Y, and D140H induced changes in the native conformation of the CDK4 protein in some behaviors and are therefore expected to have a negative impact on the protein’s role and structure.

However, the main objective of this research is to deduce the relationship between the nsSNPs and their impact on the protein-protein interaction, stability, folding and catalytic function analysis. After a concomitant structural and sequence-based study, we found the G15S mutants as the strongest malignant mutant among the three suggested mutants. Besides the *in silico* study only focuses on the broad collection of useful data which is not a definitive conclusion. Therefore, more and more experimental studies are required to confirm these computational findings. However, the present study will aid in shortening the expensive experimental cost of identifying disease-associated SNPs in CDK4 protein as we have computationally sort listed the SNPs. So, these final three mutants (G15S, D140Y and D140H) specifically G15S could be a malignant nsSNPs which was the aim of this research. So, findings of this study could be a blueprint for malignant nsSNPs identification in CDK4 gene for further clinical and experimental-based study. We believe that this study will help to differentiate malignant SNPs associated with CDK4 altered patients in future genome association studies.

## Supporting information

S1 Fig ConSurf analysis of human CDK4 protein. (DOCX)

S2 Fig Ramachandran plot analysis of (a) Mutant G15S (b) D140Y and (c) D140H protein. (DOCX)

S1 Table List of SNPs predicted by six different webservers. (S = score; DL = Deleterious; DG = Damaging; E = Effect; P = Pathogenic; N = Neutral; T = Tolerated and U = Unknown).
(DOCX)

S2 Table List of eight highest malignant nsSNPs based on compared prediction score of six different servers. (S = Score; E = Effect; DL = Deleterious; DG = Damaging; P = Pathogenic, T = Tolerated and U = Unknown).
(DOCX)

S3 Table Prediction of post translational modification sites of eight nsSNPs in CDK4 protein. (DOCX)

S4 Table Stability prediction of eight nsSNPs on CDK4 protein by I-mutant and MU-Pro webserver. (DOCX)

S5 Table Comparison analysis of predicted mutant with somatic mutants in the same codon position (S = Score; E = Effect; DL = Deleterious; DG = Damaging; P = Pathogenic, D = Decrease and LD = Large Decrease, I = Increase). (DOCX)
