# Supplementary figures and images for: Computational and structural based approach to identify malignant nonsynonymous single nucleotide polymorphisms associated with CDK4 gene
Source: PLoS One. 2021 Nov 4;16(11):e0259691. doi: 10.1371/journal.pone.0259691 (PMC8568134; doi:10.1371/journal.pone.0259691)

**
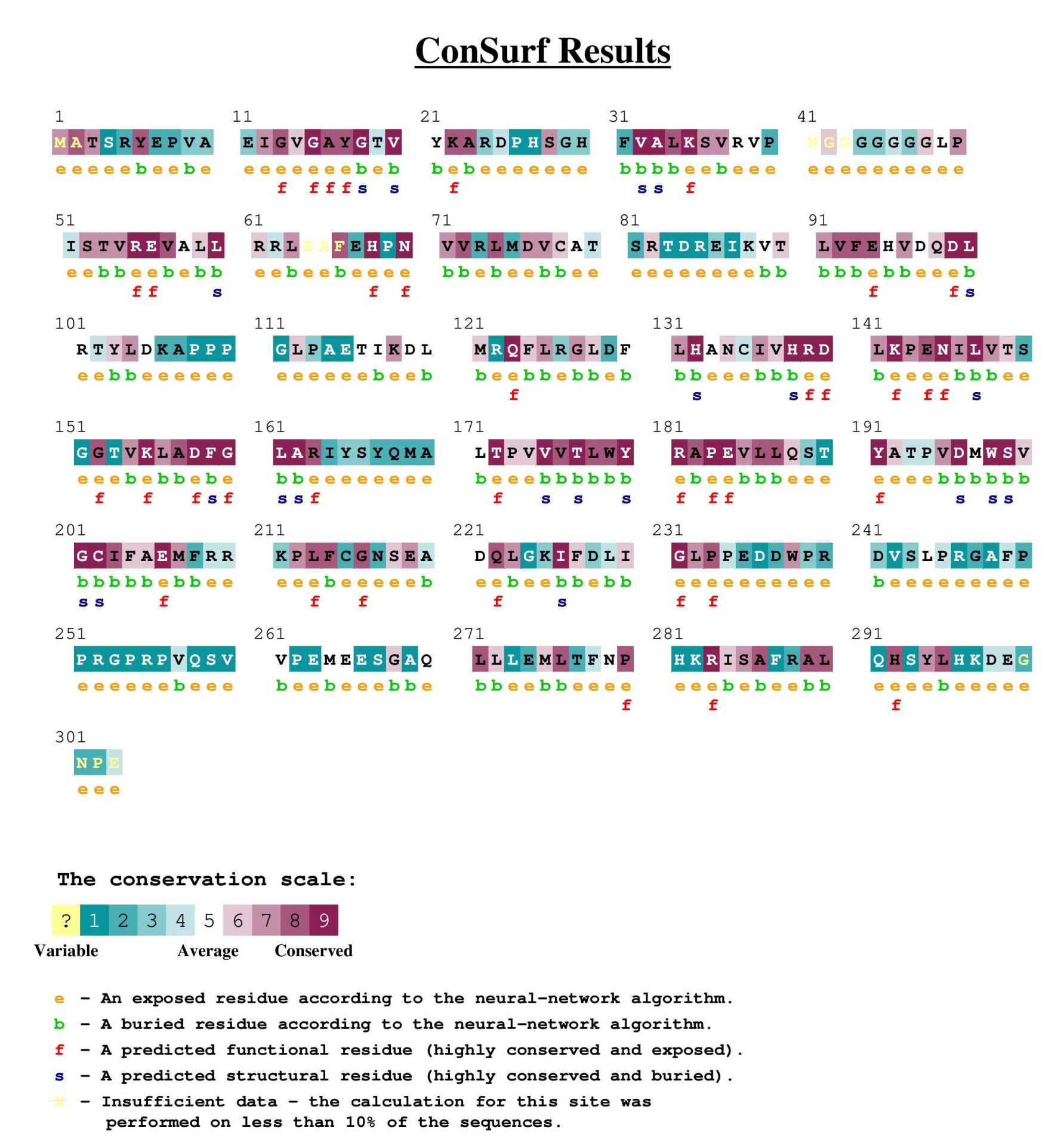
**

**S1 Fig. ConSurf analysis of human CDK4 protein**

Supplement: S1 Fig — (DOCX) [file pone.0259691.s001.docx]

**
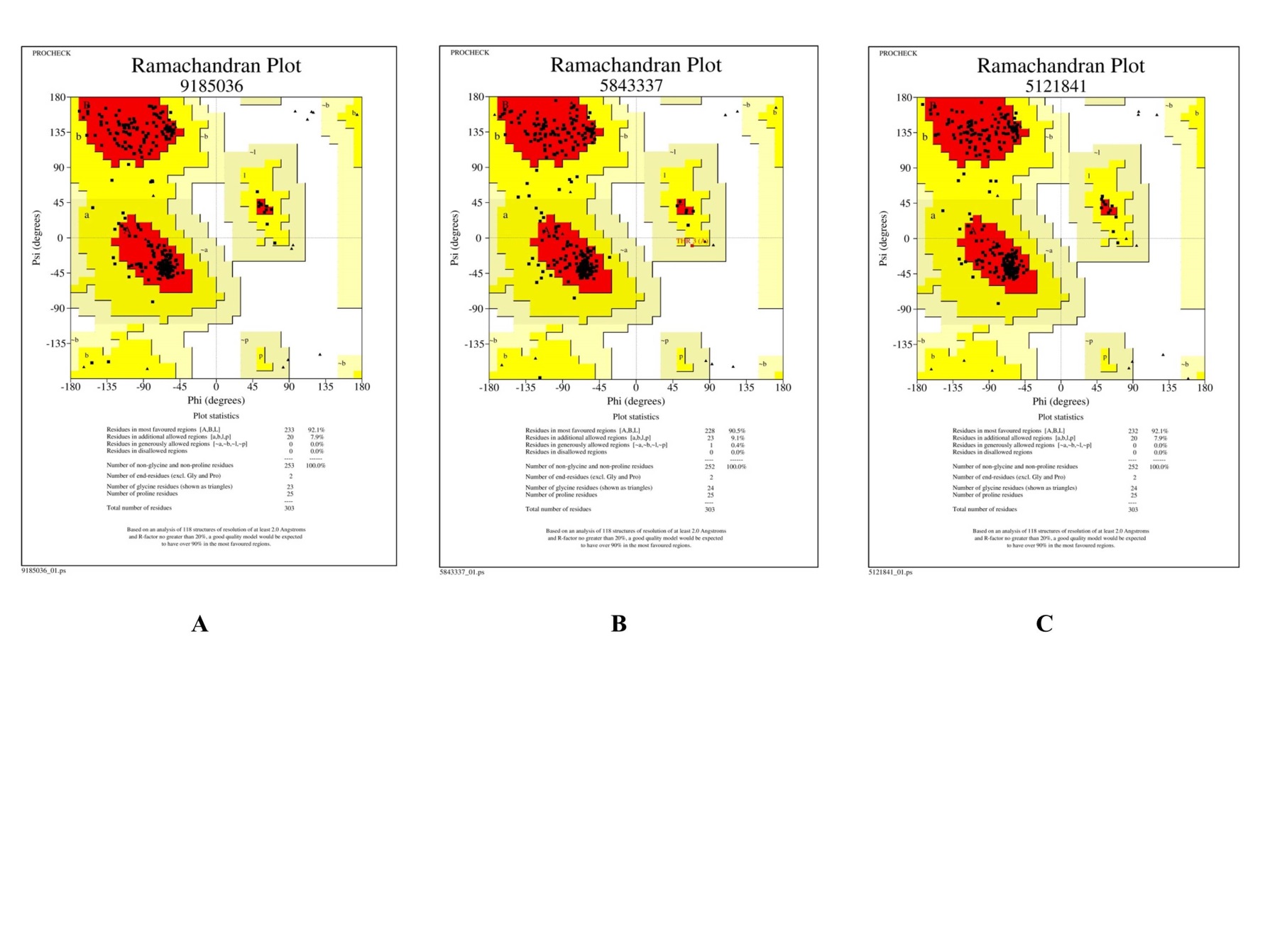
**

**S2 Fig. Ramachandran plot analysis of (a) Mutant G15S (b) D140Y and (c) D140H protein**

Supplement: S2 Fig — (DOCX) [file pone.0259691.s002.docx]
