# Supplementary material for: Computational and structural based approach to identify malignant nonsynonymous single nucleotide polymorphisms associated with CDK4 gene
Source: PLoS One. 2021 Nov 4;16(11):e0259691. doi: 10.1371/journal.pone.0259691 (PMC8568134; doi:10.1371/journal.pone.0259691)
Supplement: S1 Table — (S = score; DL = Deleterious; DG = Damaging; E = Effect; P = Pathogenic; N = Neutral; T = Tolerated and U = Unknown). (DOCX) [file pone.0259691.s003.docx]

**S1 Table. List of SNPs predicted by six different webservers.** (S=score; DL= Deleterious; DG=Damaging; E= Effect; P=Pathogenic; N=Neutral; T=Tolerated and U=Unknown)

| **dbSNP ID** | **Substitution** | **SIFT** | **S** | **PROVEAN** | **S** | **SNAP-2** | **S** | **FATHMM** | **S** | **PONP-2** | **S** | **Predict SNP** | **Confidence S** |
| --- | --- | --- | --- | --- | --- | --- | --- | --- | --- | --- | --- | --- | --- |
| rs1412237414 | H132L | DG | 0 | DL | -11.0 | E | 84 | DG | -1.9 | U | 0.60 | DL | 0.87 |
| rs868412624 | P183L | DG | 0 | DL | -9.9 | E | 69 | DG | -1.7 | U | 0.68 | DL | 0.87 |
| rs1424791303 | C202S | DG | 0 | DL | -9.7 | E | 65 | T | 0.5 | U | 0.66 | DL | 0.87 |
| rs1555201308 | D140Y | DG | 0 | DL | -8.9 | E | 94 | DG | -3.2 | U | 0.70 | DL | 0.87 |
| rs753152604 | G13V | DG | 0 | DL | -8.0 | E | 88 | DG | -1.7 | P | 0.85 | DL | 0.87 |
| rs781527596 | Y17C | DG | 0 | DL | -8.0 | E | 59 | T | 0.7 | P | 0.92 | DL | 0.87 |
| rs1223134958 | F159C | DG | 0 | DL | -8.0 | E | 78 | T | -0.9 | P | 0.77 | DL | 0.87 |
| rs1307245862 | P212H | DG | 0 | DL | -7.9 | E | 57 | T | 0.7 | U | 0.75 | DL | 0.87 |
| rs752767372 | Y103N | DG | 0 | DL | -7.8 | E | 69 | T | -0.3 | P | 0.79 | DL | 0.72 |
| rs1555201372 | R55C | DG | 0 | DL | -7.6 | E | 23 | T | 0.6 | P | 0.94 | DL | 0.87 |
| rs867386372 | P50S | DG | 0 | DL | -7.5 | E | 21 | T | -0.1 | U | 0.72 | DL | 0.61 |
| rs762519743 | P262R | DG | 0.02 | DL | -7.4 | E | 15 | T | 0.9 | P | 0.91 | DL | 0.51 |
| rs1595110706 | P108H | DG | 0.02 | DL | -7.1 | E | 21 | T | 0.9 | U | 0.76 | DL | 0.61 |
| rs1595110257 | R181G | DG | 0 | DL | -6.9 | E | 61 | T | 0.7 | U | 0.64 | DL | 0.87 |
| rs1555201308 | D140H | DG | 0 | DL | -6.9 | E | 93 | DG | -3.2 | U | 0.52 | DL | 0.87 |
| rs753579957 | R139G | DG | 0 | DL | -6.9 | E | 47 | T | -0.1 | U | 0.55 | DL | 0.87 |
| rs1565805206 | Y294C | DG | 0 | DL | -6.9 | E | 49 | T | 0.5 | P | 0.82 | DL | 0.87 |
| rs1336539869 | G13R | DG | 0 | DL | -6.9 | E | 89 | DG | -1.6 | P | 0.84 | DL | 0.87 |
| rs1555201372 | R55G | DG | 0 | DL | -6.7 | E | 69 | T | 0.6 | P | 0.82 | DL | 0.87 |
| rs1358878722 | P262T | T | 0.11 | DL | -6.6 | N | -46 | T | 0.9 | P | 0.82 | N | 0.74 |
| rs1481057693 | F227C | DG | 0.01 | DL | -6.5 | N | -11 | T | 0.9 | U | 0.67 | DL | 0.61 |
| rs1228840061 | G201D | DG | 0 | DL | -6.4 | E | 89 | DG | -2.2 | U | 0.62 | DL | 0.87 |
| rs11547328 | R24C | DG | 0 | DL | -6.4 | E | 79 | T | 0.8 | P | 0.94 | DL | 0.51 |
| rs1437365423 | R73W | DG | 0 | DL | -6.4 | E | 60 | T | 0.8 | P | 0.93 | DL | 0.61 |
| rs768127244 | L290P | DG | 0 | DL | -6.3 | E | 76 | T | 0.4 | P | 0.95 | DL | 0.87 |
| rs1276155314 | D221V | DG | 0 | DL | -6.2 | E | 74 | T | -0.3 | U | 0.82 | DL | 0.76 |
| rs1595110816 | C78S | DG | 0.01 | DL | -6.2 | E | 23 | T | 1.0 | U | 0.81 | N | 0.83 |
| rs1198423952 | P194L | DG | 0 | DL | -6.0 | E | 30 | T | -0.2 | U | 0.57 | DL | 0.51 |
| rs1565806567 | H132Y | DG | 0 | DL | -6.0 | E | 80 | DG | -1.8 | U | 0.39 | DL | 0.87 |
| rs1565806506 | A162D | DG | 0 | DL | -5.8 | E | 53 | T | -0.1 | U | 0.71 | DL | 0.87 |
| rs1299577422 | P280S | DG | 0.04 | DL | -5.7 | E | 28 | T | 0.4 | U | 0.71 | DL | 0.55 |
| rs955815089 | P108S | T | 0.17 | DL | -5.7 | N | -54 | T | 1.0 | U | 0.74 | N | 0.83 |
| rs1487430935 | A157D | DG | 0 | DL | -5.7 | E | 36 | T | 0.7 | U | 0.31 | DL | 0.87 |
| rs1355460580 | G15S | DG | 0 | DL | -5.6 | E | 87 | DG | -4.0 | P | 0.86 | DL | 0.87 |
| rs1555201131 | F214L | T | 0.06 | DL | -5.6 | E | 59 | T | 0.6 | U | 0.63 | N | 0.60 |
| rs104894340 | R24L | DG | 0 | DL | -5.6 | N | -34 | T | 1.0 | P | 0.84 | N | 0.63 |
| rs763429253 | G152E | DG | 0.01 | DL | -5.3 | E | 46 | T | 0.7 | P | 0.88 | DL | 0.76 |
| rs766166813 | G111A | T | 0.21 | DL | -5.2 | E | 9 | T | -0.1 | U | 0.69 | N | 0.75 |
| rs1236598333 | D119V | DG | 0.05 | DL | -5.1 | E | 10 | T | -0.2 | P | 0.77 | N | 0.83 |
| rs1368528782 | F66S | T | 0.25 | DL | -5.1 | N | -72 | T | -0.9 | P | 0.82 | N | 0.68 |
| rs1277385976 | G111S | T | 0.21 | DL | -5.0 | N | -10 | T | -0.1 | U | 0.73 | N | 0.74 |
| rs1478574297 | F93L | DG | 0 | DL | -5.0 | E | 23 | T | 0.0 | U | 0.49 | N | 0.60 |
| rs1555201280 | T177A | DG | 0.03 | DL | -5.0 | E | 44 | T | -0.3 | U | 0.32 | DL | 0.87 |
| rs1565806427 | L178R | DG | 0.05 | DL | -5.0 | E | 39 | T | 1.0 | U | 0.63 | DL | 0.61 |
| rs375372343 | Y180H | DG | 0 | DL | -5.0 | E | 27 | T | -0.6 | U | 0.71 | DL | 0.76 |
| rs1324120646 | Q222H | DG | 0 | DL | -4.9 | E | 50 | T | 0.8 | U | 0.62 | DL | 0.76 |
| rs759017803 | R85W | DG | 0.03 | DL | -4.9 | E | 44 | T | 0.9 | U | 0.49 | DL | 0.72 |
| rs1555201301 | A162P | DG | 0 | DL | -4.9 | E | 34 | T | -0.1 | U | 0.60 | DL | 0.87 |
| rs373619077 | R210P | T | 0.06 | DL | -4.9 | E | 71 | T | -0.2 | P | 0.84 | DL | 0.76 |
| rs1228267701 | N279Y | DG | 0.02 | DL | -4.6 | N | -2 | T | 0.8 | U | 0.48 | DL | 0.61 |
| rs1234167326 | P256L | T | 0.2 | DL | -4.6 | E | 15 | T | 1.0 | U | 0.63 | N | 0.65 |
| rs1339920302 | M207K | DG | 0 | DL | -4.5 | E | 57 | T | 0.6 | P | 0.79 | DL | 0.87 |
| rs768477694 | L49P | DG | 0.01 | DL | -4.5 | E | 46 | T | -0.2 | P | 0.87 | DL | 0.76 |
| rs587778185 | R122C | DG | 0.02 | DL | -4.5 | N | -21 | T | 0.9 | U | 0.70 | N | 0.83 |
| rs760719270 | S52I | DG | 0 | DL | -4.5 | E | 19 | T | -0.2 | P | 0.77 | DL | 0.72 |
| rs1555201383 | Y17H | DG | 0 | DL | -4.5 | E | 23 | T | 0.7 | U | 0.69 | DL | 0.87 |
| rs1060501932 | D97H | DG | 0.03 | DL | -4.4 | N | -38 | T | 0.8 | U | 0.53 | N | 0.60 |
| rs543769986 | T193P | T | 0.12 | DL | -4.4 | E | 35 | T | -0.2 | U | 0.54 | N | 0.63 |
| rs863224603 | E144G | DG | 0 | DL | -4.3 | E | 59 | T | 0.8 | U | 0.43 | N | 0.75 |
| rs11547328 | R24S | DG | 0 | DL | -4.3 | E | 31 | T | 0.9 | P | 0.87 | DL | 0.65 |
| rs1595110952 | N70S | DG | 0 | DL | -4.3 | E | 33 | T | 0.5 | U | 0.63 | DL | 0.55 |
| rs876658462 | R163S | DG | 0 | DL | -4.3 | E | 57 | T | -0.2 | U | 0.72 | DL | 0.87 |
| rs876660217 | S243Y | T | 0.07 | DL | -4.2 | N | -15 | T | -0.3 | U | 0.55 | N | 0.63 |
| rs763984661 | L229P | DG | 0.02 | DL | -4.2 | E | 64 | T | -0.2 | P | 0.85 | DL | 0.87 |
| rs876660606 | D129N | T | 0.2 | DL | -4.2 | N | -85 | T | -0.1 | U | 0.51 | N | 0.83 |
| rs200215596 | V260E | T | 0.21 | DL | -4.1 | N | -14 | T | 0.9 | U | 0.65 | N | 0.63 |
| rs1193246679 | F227L | DG | 0.01 | DL | -4.0 | E | 32 | T | 1.0 | U | 0.56 | N | 0.83 |
| rs587778185 | R122G | DG | 0.03 | DL | -4.0 | N | -24 | T | 0.9 | U | 0.66 | N | 0.74 |
| rs772079285 | R181Q | DG | 0 | DL | -4.0 | E | 72 | T | 0.7 | U | 0.63 | DL | 0.87 |
| rs1595110227 | E184K | DG | 0 | DL | -4.0 | E | 41 | T | -0.5 | U | 0.71 | DL | 0.87 |
| rs763652580 | R139Q | DG | 0 | DL | -3.9 | E | 56 | T | -0.1 | U | 0.72 | DL | 0.87 |
| rs104894340 | R24H | DG | 0 | DL | -3.9 | E | 91 | T | 0.8 | P | 0.86 | DL | 0.51 |
| rs748983604 | M75R | DG | 0 | DL | -3.9 | N | -43 | T | -0.1 | U | 0.70 | N | 0.75 |
| rs1264069202 | P194T | DG | 0.03 | DL | -3.9 | N | -60 | T | -0.2 | U | 0.29 | N | 0.83 |
| rs1595107806 | R283Q | DG | 0 | DL | -3.9 | E | 90 | T | -0.9 | U | 0.74 | DL | 0.87 |
| rs1060501929 | V200G | DG | 0 | DL | -3.8 | E | 38 | T | -0.3 | U | 0.51 | DL | 0.87 |
| rs1339920302 | M207T | DG | 0 | DL | -3.8 | E | 24 | T | 0.6 | P | 0.79 | DL | 0.76 |
| rs1595111239 | E11G | T | 0.29 | DL | -3.8 | E | 51 | T | -0.2 | U | 0.42 | N | 0.65 |
| rs1180557312 | V72A | DG | 0 | DL | -3.7 | E | 52 | T | 0.4 | U | 0.64 | DL | 0.87 |
| rs1555201108 | D237A | T | 0.68 | DL | -3.7 | N | -14 | T | -0.2 | U | 0.34 | N | 0.83 |
| rs1030964946 | L178F | DG | 0 | DL | -3.6 | E | 67 | T | 0.8 | U | 0.40 | DL | 0.87 |
| rs755549181 | V20A | DG | 0 | DL | -3.6 | E | 22 | T | -0.5 | U | 0.64 | DL | 0.87 |
| rs765656720 | K211M | DG | 0.02 | DL | -3.6 | E | 26 | T | -0.3 | U | 0.52 | DL | 0.51 |
| rs1425071781 | V32A | DG | 0 | DL | -3.5 | E | 45 | T | -0.6 | U | 0.65 | DL | 0.87 |
| rs1060501932 | D97N | T | 0.07 | DL | -3.5 | N | -33 | T | 0.9 | U | 0.59 | N | 0.83 |
| rs368013594 | A205T | DG | 0 | DL | -3.5 | N | -23 | T | -0.3 | U | 0.43 | N | 0.60 |
| rs1555201304 | V154A | DG | 0 | DL | -3.5 | E | 38 | T | 0.8 | U | 0.64 | DL | 0.87 |
| rs759017803 | R85G | T | 0.21 | DL | -3.4 | N | -15 | T | 1.0 | U | 0.47 | N | 0.83 |
| rs1403703683 | D236V | T | 0.06 | DL | -3.3 | N | -33 | T | -0.1 | U | 0.47 | N | 0.74 |
| rs1555201381 | K22Q | DG | 0.02 | DL | -3.3 | E | 33 | T | -0.3 | P | 0.77 | DL | 0.61 |
| rs587778188 | R255C | DG | 0.03 | DL | -3.3 | N | -4 | T | 0.9 | U | 0.46 | N | 0.60 |
| rs765656720 | K211T | T | 0.17 | DL | -3.2 | N | -49 | T | -0.3 | U | 0.37 | N | 0.74 |
| rs1264069202 | P194S | T | 0.54 | DL | -3.2 | N | -76 | T | -0.1 | N | 0.16 | N | 0.83 |
| rs748983604 | M75T | DG | 0.01 | DL | -3.1 | N | -33 | T | -0.1 | P | 0.78 | N | 0.63 |
| rs140644696 | R209C | DG | 0.02 | DL | -3.1 | N | -69 | T | 1.0 | U | 0.77 | N | 0.74 |
| rs975342748 | M197T | DG | 0.02 | DL | -3.0 | N | -8 | T | -0.2 | U | 0.53 | DL | 0.55 |
| rs1595111197 | T19A | T | 0.06 | DL | -3.0 | N | -35 | T | -0.2 | U | 0.54 | N | 0.74 |
| rs760435132 | I230T | DG | 0 | DL | -3.0 | N | -48 | T | 0.9 | U | 0.61 | N | 0.75 |
| rs1000052939 | E7K | DG | 0.02 | DL | -2.9 | N | -25 | T | -0.2 | U | 0.67 | N | 0.63 |
| rs1595110530 | N134S | DG | 0.02 | DL | -2.8 | E | 23 | T | -0.2 | U | 0.34 | N | 0.63 |
| rs771702415 | P234L | T | 0.06 | DL | -2.8 | N | -44 | T | 0.9 | U | 0.48 | N | 0.74 |
| rs1555201301 | A162S | DG | 0.05 | DL | -2.8 | E | 1 | T | 0.3 | U | 0.37 | N | 0.60 |
| rs1246306482 | L141V | DG | 0 | DL | -2.8 | N | 0 | T | 0.2 | U | 0.48 | DL | 0.87 |
| rs1297848043 | V20L | DG | 0 | DL | -2.8 | E | 32 | T | -0.5 | U | 0.57 | DL | 0.76 |
| rs751365131 | I284T | DG | 0.02 | DL | -2.7 | E | 26 | T | -0.3 | U | 0.64 | N | 0.60 |
| rs1595107784 | A286S | DG | 0.01 | DL | -2.7 | N | -43 | T | -0.4 | U | 0.38 | DL | 0.51 |
| rs370258992 | R246C | DG | 0 | DL | -2.7 | E | 19 | T | 0.9 | U | 0.61 | N | 0.74 |
| rs1222529576 | I164S | T | 0.07 | DL | -2.7 | N | -86 | T | -0.1 | P | 0.78 | N | 0.74 |
| rs370258992 | R246G | DG | 0.03 | DL | -2.6 | E | 17 | T | 1.0 | U | 0.51 | N | 0.83 |
| rs780052789 | V174M | DG | 0.03 | DL | -2.6 | E | 26 | T | 0.9 | U | 0.36 | N | 0.60 |
| rs373619077 | R210Q | T | 0.29 | DL | -2.6 | N | -51 | T | -0.1 | U | 0.62 | N | 0.74 |
| rs1595110684 | L112F | DG | 0.01 | DL | -2.5 | N | -15 | T | -0.4 | U | 0.37 | N | 0.68 |
