# Supplementary material for: Computational and structural based approach to identify malignant nonsynonymous single nucleotide polymorphisms associated with CDK4 gene
Source: PLoS One. 2021 Nov 4;16(11):e0259691. doi: 10.1371/journal.pone.0259691 (PMC8568134; doi:10.1371/journal.pone.0259691)
Supplement: S2 Table — (S = Score; E = Effect; DL = Deleterious; DG = Damaging; P = Pathogenic, T = Tolerated and U = Unknown). (DOCX) [file pone.0259691.s004.docx]

**S2 Table. List of eight highest malignant nsSNPs based on compared prediction score of six different servers.** (S=Score; E=Effect; DL=Deleterious; DG= Damaging; P=Pathogenic, T=Tolerated and U= Unknown).

| **Substitution** | **SIFT** | **S** | **PROVEAN** | **S** | **SNAP-2** | **S** | **FATHMM** | **S** | **PONP-2** | **S** | **Predict SNP** | **Confidence S** |
| --- | --- | --- | --- | --- | --- | --- | --- | --- | --- | --- | --- | --- |
| G15S | DG | 0 | DL | -5.6 | E | 87 | DG | -3.98 | P | 0.86 | DL | 0.87 |
| D140Y | DG | 0 | DL | -8.88 | E | 94 | DG | -3.16 | U | 0.70 | DL | 0.87 |
| G13R | DG | 0 | DL | -6.88 | E | 89 | DG | -1.64 | P | 0.84 | DL | 0.87 |
| G13V | DG | 0 | DL | -8 | E | 88 | DG | -1.65 | P | 0.85 | DL | 0.87 |
| H132L | DG | 0 | DL | -10.96 | E | 84 | DG | -1.85 | U | 0.60 | DL | 0.87 |
| P183L | DG | 0 | DL | -9.91 | E | 69 | DG | -1.66 | U | 0.68 | DL | 0.87 |
| G201D | DG | 0 | DL | -6.44 | E | 89 | DG | -2.22 | U | 0.62 | DL | 0.87 |
| D140H | DG | 0 | DL | -6.91 | E | 93 | DG | -3.16 | U | 0.52 | DL | 0.87 |
