# Supplementary material for: Computational and structural based approach to identify malignant nonsynonymous single nucleotide polymorphisms associated with CDK4 gene
Source: PLoS One. 2021 Nov 4;16(11):e0259691. doi: 10.1371/journal.pone.0259691 (PMC8568134; doi:10.1371/journal.pone.0259691)
Supplement: S3 Table — (DOCX) [file pone.0259691.s005.docx]

**S3 Table. Prediction of post translational modification sites of eight nsSNPs in CDK4 protein**

| **SNP ID** | **AA change** | **PTM (Using ModPred- sequence based prediction method)** | |
| --- | --- | --- | --- |
|  | | **Modification** | **Score** |
| rs1355460580 | G15S | Proteolytic cleavage | 0.68 |
| rs1555201308 | D140Y | Proteolytic cleavage | 0.73 |
| rs1336539869 | G13R | - | - |
| rs753152604 | G13V | - | - |
| rs1412237414 | H132L | Proteolytic cleavage | 0.53 |
| rs868412624 | P183L | - | - |
| rs1228840061 | G201D | - | - |
| rs1555201308 | D140H | Proteolytic cleavage | 0.73 |
