# Supplementary material for: Computational and structural based approach to identify malignant nonsynonymous single nucleotide polymorphisms associated with CDK4 gene
Source: PLoS One. 2021 Nov 4;16(11):e0259691. doi: 10.1371/journal.pone.0259691 (PMC8568134; doi:10.1371/journal.pone.0259691)
Supplement: S4 Table — (DOCX) [file pone.0259691.s006.docx]

**S4 Table. Stability prediction of eight nsSNPs on CDK4 protein by I-mutant and MU-Pro webserver**

| **dbSNP ID** | **Substitution** | **I-MUTANT** | **DDG Value** | **MU-PRO** | **delG** |
| --- | --- | --- | --- | --- | --- |
| rs1355460580 | G15S | Large Decrease | -1.23 | Decrease | -0.08 |
| rs1555201308 | D140Y | Large Decrease | -0.57 | Decrease | -0.77 |
| rs1336539869 | G13R | Decrease | -0.11 | Decrease | -0.42 |
| rs753152604 | G13V | Increase | 0.02 | Decrease | -0.32 |
| rs1412237414 | H132L | Increase | 0.19 | Increase | 0.2 |
| rs868412624 | P183L | Increase | 0.09 | Increase | 0.11 |
| rs1228840061 | G201D | Decrease | -0.3 | Decrease | -0.37 |
| rs1555201308 | D140H | Large Decrease | -0.63 | Decrease | -1.2 |
