# Supplementary material for: Computational and structural based approach to identify malignant nonsynonymous single nucleotide polymorphisms associated with CDK4 gene
Source: PLoS One. 2021 Nov 4;16(11):e0259691. doi: 10.1371/journal.pone.0259691 (PMC8568134; doi:10.1371/journal.pone.0259691)
Supplement: S5 Table — (DOCX) [file pone.0259691.s007.docx]

**S5 Table. Comparison analysis of predicted mutant with somatic mutants in the same codon position** (S=Score; E=Effect; DL=Deleterious; DG= Damaging; P=Pathogenic, D=Decrease and LD= Large Decrease, I=Increase)

| **Mutation ID** | **Substitution** | **SIFT** | **S** | **PROVEAN** | **S** | **SNAP-2** | **S** | **FATHMM** | **S** | **PONP-2** | **S** | **Predict SNP** | **S** | **I Mutant** | **S** | **Mu Pro** | **S** |
| --- | --- | --- | --- | --- | --- | --- | --- | --- | --- | --- | --- | --- | --- | --- | --- | --- | --- |
| COSM8456275 | G15R | DG | 0 | DL | -7.46 | E | 81 | DG | -3.9 | P | 0.93 | DL | 0.87 | D | -0.95 | D | -0.05 |
| COSM8030831 | G15C | DG | 0 | DL | -8.31 | E | 14 | DG | -3.9 | P | 0.82 | DL | 0.87 | D | -0.73 | I | 0.18 |
| rs1355460580 | G15S | DG | 0 | DL | -5.6 | E | 87 | DG | -3.9 | P | 0.85 | DL | 0.86 | LD | -1.23 | D | -0.08 |
